# Landscape and evolutionary dynamics of terminal repeat retrotransposons in miniature in plant genomes

**DOI:** 10.1186/s13059-015-0867-y

**Published:** 2016-01-18

**Authors:** Dongying Gao, Yupeng Li, Kyung Do Kim, Brian Abernathy, Scott A. Jackson

**Affiliations:** Center for Applied Genetic Technologies, University of Georgia, 111 Riverbend Road, Athens, GA 30602 USA

**Keywords:** Gene evolution, Genomics, Plant, Retrotransposition, TRIM

## Abstract

**Background:**

Terminal repeat retrotransposons in miniature (TRIMs) are a unique group of small long terminal repeat retrotransposons that are difficult to identify. Thus far, only a few TRIMs have been characterized in the euphyllophytes, and their evolutionary and biological significance as well as their transposition mechanisms are poorly understood.

**Results:**

Using a combination of de novo and homology-based methods, we annotate TRIMs in 48 plant genome sequences, spanning land plants to algae. The TRIMs are grouped into 156 families including 145 that were previously undefined. Notably, we identify the first TRIMs in a lycophyte and non-vascular plants. The majority of the TRIM families are highly conserved and shared within and between plant families. Unlike other long terminal repeat retrotransposons, TRIMs are enriched in or near genes; they are also targeted by sRNAs between 21 and 24 nucleotides in length, and are frequently found in CG body-methylated genes. Importantly, we also identify putative autonomous retrotransposons and very recent transpositions of a TRIM element in *Oryza sativa*.

**Conclusions:**

We perform the most comprehensive analysis of TRIM transposons thus far and report that TRIMs are ubiquitous across plant genomes. Our results show that TRIMs are more frequently associated with large and CG body-methylated genes that have undergone strong purifying selection. Our findings also indicate that TRIMs are likely derived from internal deletions of large long terminal repeat retrotransposons. Finally, our data and methodology are important resources for the characterization and evolutionary and genomic studies of long terminal repeat retrotransposons in other genomes.

**Electronic supplementary material:**

The online version of this article (doi:10.1186/s13059-015-0867-y) contains supplementary material, which is available to authorized users.

## Background

Retrotransposons are ubiquitous components of most eukaryotic genomes. These elements use an element-encoded mRNA as the transposition intermediate and can rapidly proliferate in copy number, resulting in large differences in genome sizes between related species [1, 2]. Retrotransposon-induced mutations are usually stable and are used as molecular tools for gene-tagging and functional analysis [3]. Retroelements can provide raw material for evolutionary innovation, including new genes and gene regulatory networks [4]. Furthermore, retroelements can form functional genomic elements that regulate gene expression, maintain chromatin structure, and contribute to histone modification and DNA methylation [5, 6].

Long terminal repeat (LTR) retrotransposons are the most abundant mobile elements in the plant kingdom. For example, there are more than 1.1 million LTR retroelements in maize, accounting for 75 % of the genome [7]. LTR retrotransposons in plants can be large, up to 20 kilobases (kb), and have LTRs more than 5 kb in length [1]. These elements are often clustered into blocks that can exceed 100 kb via layers of nested insertions [8]. Moreover, LTR retrotransposons can have distinct chromosomal distribution patterns. For example, LTR retrotransposons can be found in intergenic regions but are most often concentrated in highly heterochromatic regions [9–11]. Plant LTR retrotransposons are very dynamic and with only a few exceptions, for example, centromeric retrotransposons in grasses [2, 12], are not conserved at the sequence level between related species.

Terminal repeat retrotransposons in miniature (TRIMs) maintain some similarities with LTR retrotransposons, including terminal direct repeats and target site duplication (TSD) of 4–6 bp, but they are small, less than 1,000 bp [13, 14] and as small as 292 bp [15], and do not encode the retrotransposon proteins needed for movement, such as reverse transcriptase, integrase, and others. Owing to their extremely short length and lack of capacity to encode proteins, TRIMs are difficult to annotate. To date, only 11 TRIM families, *Katydid*-At1, At2, At3 [14], Br1–Br4, *Katydid*-At4 [16], Cassandra [17, 18], SMART [15], and Wukong [13], have been reported in the euphyllophytes. Recently, a TRIM was reported in the red harvester ant (*Pogonomyrmex barbatus*, PbTRIM) [19], the only one reported in animals. Most of these studies have focused on one or a few TRIM families and no TRIM elements have been found in lycophytes or non-vascular plants. Thus, the evolutionary impacts of TRIMs on host genomes and the mechanisms involved in their emergence and disappearance remain poorly understood. Owing to the availability of more plant genome sequences, we are now able to analyze and compare TRIMs across a broad evolutionary range of species.

To understand the evolution and mobility of TRIMs, we analyzed 48 genome sequences, including spermatophytes (seed plants), lycophyte, bryophytes, and algae. We identified complete TRIM elements in all the flowering plants and, for first time, in a lycophyte and non-vascular plants. The TRIMs were grouped into 156 families, of which 145 had not previously been described. We observed that TRIMs are enriched in genic regions and likely play a role in gene evolution. TRIMs were also targeted by various sRNAs and frequently associated with CG body-methylated genes. Importantly, we identified the first putative autonomous LTR retrotransposons for a TRIM and uncovered recent transposition of a TRIM family in *Oryza sativa*. These results provide a better understanding of the dynamics and role that TRIM elements play in plant genome and gene evolution.

## Results

### Characterization and unusual organization of TRIMs

#### Identification and abundance of plant TRIMs

To annotate TRIMs in plant, we first analyzed 48 plant genomes available as of 1 April 2013 (Additional file 1: Table S1) [7, 20–65] using LTR_FINDER [66]. A total of 29,779 potential TRIM sequences were found in the 48 genomes with an average of 620 predicted sequences per genome. The minimum number of annotated sequences predicted for a single genome was 16 in *Thellungiella parvula* [43], and the maximum number was 3,300 for *Ricinus communis* [35]. The 29,779 sequences were then manually inspected for structures using BLASTN and BLASTX. From this, 3,549 sequences were determined to be TRIMs and the other 26,230 sequences were discarded. The primary constituents of the discarded fraction were tandem repeats and incomplete elements: 59 % in maize and 95 % in soybean (Additional file 1: Figure S1). The conservation of TRIM elements across species has previously been reported [14, 15, 17]. Thus, TRIM elements identified by LTR_FINDER in each genome were grouped into TRIM subfamilies rather than families. The 3,549 sequences were grouped into 217 TRIM subfamilies that included Wukong and Br4, originally identified by sequence alignments of homologous regions [13, 16]. Among the 48 plant genomes, de novo annotation identified TRIMs in 40 genomes; no TRIMs were annotated in the other eight, including *Arabidopsis thaliana,* for which five TRIMs, *Katydid*-At1, At2, At3, At4, and Cassandra, had been previously annotated by sequence alignments [14, 16, 17]. This indicates that de novo annotation does not identify all TRIMs. Therefore, all 217 identified TRIM subfamilies were used to conduct homology searches and an additional 72 subfamilies were found, including three new subfamilies in *A. thaliana*. A total of 289 TRIM subfamilies were identified in 43 genomes, including all 30 eudicots and nine monocots. Notably, TRIMs were found in the lycophyte, *Selaginella moellendorffii*, and three algae genomes, *Chlamydomonas reinhardtii*, *Volvox carteri* and *Chondrus crispus* (Table 1). To our knowledge, this is the first time that TRIMs have been reported in lycophytes and non-vascular plants. However, TRIM elements were not found in *Physcomitrella patens*, and four other algae genomes, *Chlorella variabilis, Ostreococcus lucimarinus, O. tauri*, and *Cyanidioschyzon merolae*.

**Table 1:** Summary of terminal repeat retrotransposons in miniature in 43 sequenced plant genomes

Plant genome	Genus/Family of plant	Number of TRIM subfamily				Copy number		Fraction (%)
		Shared between families	Family specific	Species specific	Total	Complete	Total	
Tomato (*Solanum lycopersicum*)	*Solanum/Solanaceae*	6	4		10	560	9,162	0.32
Currant Tomato (*Solanum pimpinellifolium*)	*Solanum*/*Solanaceae*	7	4		11	178	10,199	0.29
Potato (*Solanum tuberosum*)	*Solanum/Solanaceae*	4	5		9	451	12,473	0.46
Cucumber (*Cucumis sativus*)	*Cucumis/Cucurbitaceae*	4			4	30	2,816	0.21
Muskmelon (*Cucumis melo*)	*Cucumis/Cucurbitaceae*	3			3	44	2,072	0.09
Watermelon (*Citrullus lanatus*)	Citrullus*/Cucurbitaceae*	5			5	228	4,779	0.21
Plum blossom (*Prunus mume*)	*Prunus mume/Rosaceae*	5	2		7	83	5,719	0.47
Apple (*Malus x domestica*)	*Malus/Rosaceae*	7			7	2,043	25,835	0.74
Pear (*Pyrus bretschneideri*)	*Pyrus/Rosaceae*	6	1		7	2,286	20,092	1.26
*S*trawberry (*Fragaria vesca*)	*Fragaria*/*Rosaceae*	4			4	132	1,605	0.18
Marijuana (*Cannabis sativa*)	*Cannabis/Cannabaceae*	5		1	6	362	14,147	0.55
Lotus (*Lotus japonicus*)	*Lotus/Fabaceae*	6	2	1	9	379	7,943	0.88
Barrel medic (*Medicago truncatula*)	*Medicago/Fabaceae*	7	1		8	46	8,416	0.56
Chickpea (*Cicer arietinum*)	Cicer/*Faboideae*		2		2	102	1,499	0.21
Soybean (*Glycine max*)	*Glycine/Faboideae*	9	1	6	16	261	10,102	0.25
Pigeon pea (*Cajanus cajan*)	*Cajanus/Faboideae*	9	3	1	13	840	20,915	0.67
Barbados nut (*Jatropha curcas*)	*Jatropha/Euphorbiaceae*	5		2	7	177	3,390	0.28
Flax (*Linum usitatissimum*)	*Linum/Linaceae*	3		2	5	71	4,149	0.33
Castor bean plant (*Ricinus communis*)	*Ricinus/Euphorbiaceae*	2			2	90	385	0.02
Poplar (*Populus trichocarpa*)	*Populus/Salicaceae*	5		1	6	839	5,292	0.28
Thale cress (*Arabidopsis thaliana*)	*Arabidopsis/Brassicaceae*	5	3		8	36	876	0.09
Lyrate rockcress (*Arabidopsis lyrata*)	*Arabidopsis/Brassicaceae*	9	5		14	259	1,724	0.25
Pallus (*Thellungiella salsuginea*)	*Thellungiella*/*Brassicaceae*	9	1		10	98	1,406	0.16
Turnip mustard (*Brassica rapa*)	*Brassica/Brassicaceae*	9	1		10	269	3,030	0.26
*Eutrema parvulum (Thellungiella parvula*)	*Eutrema/Brassicaceae*	3	1		4	25	539	0.10
Papaya (*Carica papaya*)	*Carica/Caricaceae*			1	1	5	897	0.09
Cocoa (*Theobroma cacao*)	*Theobroma*/*Malvaceae*			1	1	45	360	0.03
Cotton (*Gossypium raimondii*)	*Gossypium/Malvaceae*	1	2		3	19	19,008	0.35
Grape (*Vitis vinifera*)	*Vitis/Vitaceae*	4		1	5	228	8,890	0.34
Sweet orange (*Citrus sinensis*)	*Citrus/Rutaceae*	2		1	3	30	1,180	0.09
Sorghum (*Sorghum bicolor*)	*Sorghum/Poaceae*	1	5		6	282	2,922	0.11
Maize (*Zea mays*)	*Zea/Poaceae*	1	3	3	7	1,361	9,036	0.12
Foxtail (*Setaria italica*)	*Setaria/Poaceae*	1	4		5	129	1,032	0.07
Rice *japonica* (*Oryza sativa, japonica*)	*Oryza/Poaceae*	2	9		11	379	2,911	0.18
Rice *indica* (*Oryza sativa, indica*)	*Oryza/Poaceae*	2	9		11	364	3,252	0.19
Brachyantha (*Oryza brachyantha*)	*Oryza/Poaceae*	2	8	1	11	116	1,506	0.15
Purple false brome (*Brachypodium distachyon*)	*Brachypodium/Poaceae*	1	2	2	5	75	1,685	0.15
Date palm (*Phoenix dactylifera*)	*Phoenix/Arecaceae*	2		7	9	777	13,358	0.78
Banana (*Musa acuminata*)	*Musa/Musaceae*			2	2	126	4,577	0.23
Spikemoss (*Selaginella moellendorffii*)	*Selaginella/Selaginellaceae*	2		6	8	1,177	10,158	1.19
Green alga (*Chlamydomonas reinhardtii*)	*Chlamydomonas/Chlamydomonadaceae*	1		5	6	31	1,349	0.21
Volvox (*Volvox carteri*)	*Volvox/Volvocaceae*			5	5	292	2,052	0.27
Irish moss (*Chondrus crispus*)	*Chondrus*/*Gigartinaceae*			3	3	75	422	0.09
Total		159	78	52	289			

The average size of the 289 subfamilies was 685 base pairs (bp), much smaller than typical plant LTR retroelements (4–10 kb on average) [67]. Among the 289 subfamilies, 225 (77.9 %) were smaller than 1,000 bp and 197 (68.1 %) LTRs were smaller than 250 bp (Additional file 1: Figure S2A, B).

The copy numbers of TRIMs were highly variable between genomes. The majority (65 %, 28/43) of the plant genomes harbored more than 2,000 complete or fragmented TRIMs, only six (14 %) had fewer than 1,000 TRIMs (Table 1). Most, 174 of the 289 subfamilies (60 %), had copy numbers less than 500, and about one-quarter (70/289) had copy numbers greater than 1,000 (Additional file 1: Figure S2C).

#### Conservation and comparison of TRIMs

To determine the phylogenetic distribution and group the TRIM elements, the 289 TRIM subfamilies were used to search GenBank and conduct all-by-all BLASTN searches. We found 159 subfamilies in more than two plant taxonomic families; 78 subfamilies in multiple genomes from a same plant family, termed “family-specific TRIMs”; and 52 subfamilies in only a single genome, termed “species-specific TRIMs.” Species-specific TRIMs may have homologs that were either lost, diverged in other genomes, or not represented in GenBank (Table 1).

The TRIMs from the 43 plants were then grouped into families based on sequence similarity. A total of 156 TRIM families were identified, 60 of which were shared between plant families, 44 were specific to a single plant family, and 52 were species-specific. Of these 156 families, 145 were identified for the first time. We also found new members for the previously reported TRIM families [14–17], such as complete Cassandra transposons in *Cucumis sativa* and other plants.

**Fig. 1:**
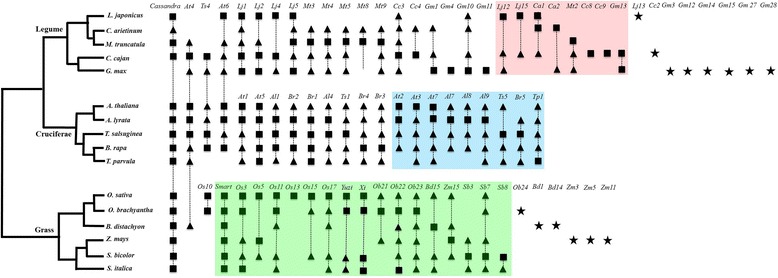
Comparison of terminal repeat retrotransposons in miniature (*TRIMs*) in three plant taxonomic families. *Black squares* and *triangles* represent complete and fragmented TRIMs, respectively, shared within and between plant genomes. *Black stars* indicate TRIMs present in a single genome. TRIMs grouped into a single family are linked by *dashed lines*. TRIMs in *pink*, *blue*, and *green* boxes are present only in legumes, Cruciferae, and grasses, respectively

The TRIMs from three plant taxonomic families, the Legumes (Fabaceae), Cruciferae (Brassicaceae), and Grasses (Poaceae), are detailed in Fig. 1. These three families were chosen as each contains more than five sequenced genomes, represents both dicots and monocots, and has ~140–150 million years (My) of evolution [68]. They provide a resource to analyze the conservation and evolution of plant TRIMs.

Within the Cruciferae, *Arabidopsis lyrata* and *Brassica rapa* shared a common ancestor with the model plant *A. thaliana* about 13 and 43 million years ago (Mya), respectively [69]. Nine TRIM families were previously reported in this plant family, including At1–4 and Cassandra in *A. thaliana* [14, 16, 17] and Br1–4 in *B. rap*a [16]. We found an additional 13 new TRIM families. Among the 22 TRIM families, two, Cassandra and At4, have complete or fragmented homologs in legumes and grasses, 11 were shared between the Cruciferae and other dicots, and nine families were found only within the Cruciferae (Fig. 1).

We found 36 TRIM families in the five legume genomes, including Cassandra and At4. Among these, 15 were shared between legumes and other plant families. Two families, GmaRetroS4 (abbreviated as *Gm4*) and GmaRetroS11 (*Gm11*) from *Glycine max*, were absent in the other four legumes but homologs were found in other plants. Eight family-specific TRIMs—LjaRetroS12 and 15, CarRetroS1 and 2, MtrRetroS2, CcaRetroS8 and 9, and GmaRetroS13—were found in subsets of the five sequenced legumes that last shared a common ancestor about 50 Mya [70].

In addition to the three previously described TRIM families—SMART [15], Cassandra [17], and Wukong [13]—we identified 22 new families within the grasses. Family OsaRetroS10 (*Os10*) had complete elements in *Oryza sativa* and *O. brachyantha* and homologs were found in *Solanum lycopersicum* (AC243477:1845–1967, E value = 7 × e^−8^) and *S. pimpinellifolium* (AGFK01075962: 4312–4434, E value = 7e^−11^). Ten TRIM families identified in *O. sativa* and *O. brachyantha* have complete and/or fragmented copies in *Zea mays* and/or *Sorghum bicolor* that diverged from the *Oryza* genus ~50–80 Mya [71].

#### Tandemly arrayed TRIMs

A typical LTR retrotransposon contains 5′ and 3′ LTRs flanking an internal region that often encodes proteins required for retrotransposition. We refer to this structure as L_2_I_1_, where L_2_ refers to two **L**TRs and I_1_ to an **I**nternal sequence. In addition to the typical TRIM elements (L_2_I_1_), some TRIMs were tandemly arranged and contained more than three LTRs and two internal regions, hereafter referred to as tandemly arrayed (TA)-TRIMs. So far, this peculiar structure has only been reported for the Cassandra TRIM, whose LTRs contain sequences similar to cellular 5S rRNA, which is also tandemly arranged [17, 18]. No 5S rRNA sequences were found in any of the other TRIM families.

**Fig. 2:**
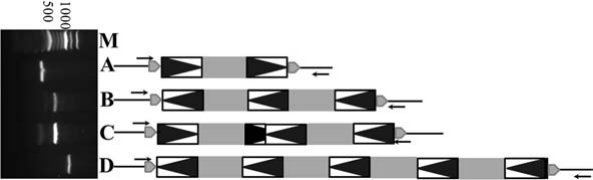
Tandemly arrayed terminal repeat retrotransposons in miniature (TA-TRIMs) of Zma-SMART in the maize genome. Boxes containing *black triangles* indicate the long terminal repeats (LTRs) of TRIMs and *gray boxes* denote the internal regions of TRIMs. The *gray pentagons* are target site duplications (TSDs) that flank TRIMs and *arrows* indicate the polymerase chain reaction primers used to validate the TRIM sequences. *M* indicates a 100 base pair DNA ladder; *A* indicates a typical Zma-SMARTTRIM with two LTRs and one internal region (AC186328:154584–154863; TSD:AACAT); *B* indicates a TA-TRIM with three LTRs and two internal regions (AC210283: 61391–61889; TSD: GGGTT); *C* indicates a TA-TRIM with two inverted TRIMs (AC220956: 117725–118283; TSD: CTTCA); and *D* indicates a TA-TRIM with five LTRs and four internal regions (AC185340: 80554–81415; TSD: ATAAT)

We found that TA-TRIMS are common in plant genomes, with 129 subfamilies having TA-TRIM structures in 35 of the 43 genomes (Additional file 1: Table S2). To gain more insight into TA-TRIMs, we focused on maize, where there were 93 tandem arrays from four TRIM subfamilies. These arrays varied in organization and contained varying numbers of LTRs and internal sequences, such as three LTRs and two internal regions (L_3_I_2_), and five LTRs and four internal regions (L_5_I_4_) (Fig. 2, Additional file 1: Table S3). Among all the TA-TRIMs identified in maize, L_3_I_2_ was the most frequent, accounting for more than 67 % (63/93) of all TA-TRIMs. To validate TA-TRIMs in maize, we conducted polymerase chain reaction (PCR) analysis using primers that targeted regions flanking TA-TRIMs from the Zma-SMART subfamily (Fig. 2), and further confirmed these structures by DNA sequencing. This validated the structure and organization of the TA-TRIMs, confirming that they were not artifacts of errors in genome assembly.

### TRIM-mediated gene evolution

#### Enrichment of TRIMs in genic regions

TRIMs have been postulated to be involved in gene divergence and regulation [14, 15, 17]. However, these studies focused on only one or a few TRIM families and did not provide a genome-wide and cross-species view of the impact of TRIMs on gene evolution and function. Therefore, we examined the distribution of TRIMs with respect to genes in 14 of the plant genomes. Our data indicate that TRIMs are enriched in genic regions, 18.8–49.4 % were located in or near (1.5 kb upstream) genes (Additional file 1: Table S4). Interestingly, an average of 2.7 % of the TRIMs within a genome have been recruited as exons, based on an analysis of annotated genes, including coding DNA sequences and untranslated regions (UTRs). In the red harvester ant, ~45 % of the TRIMs were present within or near predicted genes [19]. These results indicate that TRIMs may exhibit preferential insertion/retention in or near genes, in both plants and animals.

We further analyzed Ty1-copia and Ty3-gypsy LTR retrotransposons and miniature inverted–repeat transposable elements (MITEs) in *G. max* and *Z. mays* and compared their distributions with the annotated genes. We found that 4.1 % of Ty3 and 6.3 % of Ty1 retrotransposons were located in genic regions in *Z. mays,* and 11.7 % of Ty3 and 16.5 % of Ty1 retrotransposons were located in genic regions in *G. max* (Additional file 1: Table S5). These percentages were significant lower than TRIMs (Pearson’s Chi-squared test, *p*-value < 2.2e^−16^). MITEs are small DNA transposons that have insertion preferences in or near genes [72, 73]. We detected 37.1 % of MITEs in *Z. mays* and 37.4 % in *G. max* in and near genes, but TRIMs were present in genic regions at significantly higher frequencies in *G. max* but lower frequencies in *Z. mays* (Pearson’s Chi-squared test, *p*-value < 2.2e^−16^).

#### Insertion/maintenance in larger genes

We compared gene structures of TRIM-related genes (TRGs), genes that contain TRIM sequences, and non-TRIM-related genes (NTRGs) in *G. max* and *Z. mays*. In both genomes, TRGs had more exons and were larger than NTRGs (Additional file 1: Figure S3, Table S6). For example, in *G. max* the average exon number of TRGs was 12.2 versus 5.9 for NTRGs. Differences in exon number, exon size, and intron size between TRGs and NTRGs were statistically significant for both species: *p*-values from two-sample t-tests after log transformation were less than 2.2 × 10^−16^.

Because larger genes have more space to harbor transposable elements (TEs), we compared the density of TRIMs between larger and smaller genes to determine if the observation of TRGs being large was just an artifact of there being more space for a TRIM to insert. All annotated genes in *G. max* and *Z. mays* were ranked from smallest to largest, and the top and bottom 20 % were defined as “small” and “large” genes. We found 21 TRIMs in small (9,273 covering 7621 kb) genes and 1,554 TRIMs in large (9,273 covering 84,971 kb) genes in *G. max*. In *G. max*, the TRIM density in large genes was 0.17 insertions/gene, ~73 times higher than in small genes; on a per kbp basis, large genes were 6.5 times more likely to have TRIM insertions (0.0183 for large versus 0.0028 for small). In *Z. mays*, large genes also had a significantly higher density of TRIMs at 0.17 insertions/gene, ~53 times more than small genes (~2 times more on a per kbp basis) (Additional file 1: Table S7).

Because TRIMs are small, we expected relatively little contribution to the expansion of genes. Thus, the large differences in exon number and gene size may reflect an accumulation bias of TRIMs into larger genes. To test this hypothesis, TRGs and NTRGs in the two genomes were used to find orthologous genes in their closest relatives: *Cajanus cajan* and *Phaseolus vulgaris* for *G. max*, which diverged ~20 and 15 Mya, respectively [70]; and *S. bicolor* and *O. sativa* for *Z. mays*, which diverged ~10 and 50–80 Mya, respectively [71]. Results from all four genomes indicated that homologs of TRGs also have higher exon numbers and are larger than orthologs of NTRGs. The exon number and sizes of TRGs and NTRGs were similar to their orthologous genes (Additional file 1: Table S8). However, the introns of both TRGs and NTRGs in *Z. mays* were larger than their orthologs from *S. bicolor* and *O. sativa*, likely due to the higher transposon density in *Z. mays* [7].

To gain more insight into the distribution of TRIMs, we analyzed 30,853 genes in *G. max* and 23,670 genes in *Z. mays* that have defined syntenic orthologs in *P. vulgaris* and *S. bicolor*, respectively [74, 75]. In addition, we compared the distributions of TRIMs with Ty1 and Ty3 LTR retrotransposons and MITEs. TRIMs were significantly more frequent in genic regions than other TEs in both *G. max* and *Z. mays*, but at a lower percentage than MITEs in *Z. mays* (Additional file 1: Table S9). These results are similar to those from all annotated genes (Additional file 1: Table S5) and further support the observation that TRIMs are enriched in genic regions. We further investigated the structure of genes containing TRIMs or other TEs and found that the syntenic genes in which TRIMs served as exons or introns were significantly larger and had more exons than the genes without TRIMs in both genomes (t-test, *p*-value < 2.23^−180^). In addition, genes containing TRIMs were significantly bigger than the genes with MITEs in both genomes (Additional file 1: Table S10). Significant length differences were detected between the syntenic genes containing TRIMs and other LTR retrotransposons in *G. max*, but not in *Z. mays* (Additional file 1: Table S10). Given that the average size of Ty1 and Ty3 retrotransposons located in syntenic genes in *Z. mays* was 930.8 and 1211.9 bp, four to five times larger than TRIMs (219.9 bp), we assume that Ty1 and Ty3 retrotransposons enlarged the related genes. Taken together, these results indicate that TRIMs either preferentially insert into or are retained in large genes.

#### Purifying selection of TRIM-related genes

To explore the selective pressures that may have acted on TRGs, we calculated the ratio of the number of non-synonymous substitutions per non-synonymous site (*Ka*) to the number of synonymous substitutions per synonymous site (*Ks*) of the genes from *G. max* and *Z. mays* by conducting genome-wide pairwise comparisons with their homologous genes in *P. vulgaris* and *S. bicolor* using gKaKs [76]. In *G. max*, the average *Ka* value of TRGs was similar to that of NTRGs, but the average *Ks* value of TRGs was significantly lower than that for NTRGs (*p*-value < 2.2 × 10^−16^; Wilcoxon rank-sum test). In *Z. mays*, the average values for both *Ka* and *Ks* of TRGs were significantly lower than for NTRGs and indicated lower evolutionary rates for TRGs, consistent with our observation that TRGs are more conserved than NTRGs. It is interesting that the average *Ka*/*Ks* value of TRGs was 0.19 in *G. max* and 0.25 in *Z. mays*, much lower than 1.0 and significantly lower than that of NTRGs (*p*-value = 5.3 × 10^−07^ for *G. max*, *p*-value < 2.2 × 10^−16^ for *Z. mays*; Wilcoxon rank-sum test) (Additional file 1: Table S11). These results indicate that TRGs have likely undergone strong purifying selection.

#### Gene acquisitions related to TRIMs

**Fig. 3:**
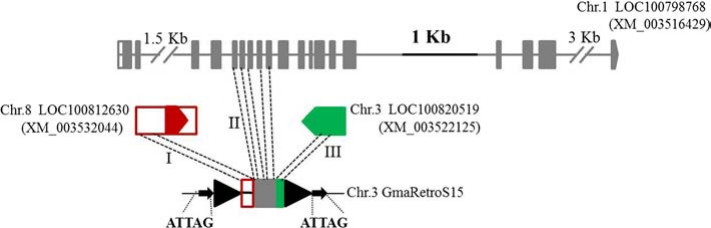
Gene acquisitions related to terminal repeat retrotransposon in miniature (*TRIM*) GmaRetroS15 in *Glycine max. Black triangles* and *arrows* denote TRIM long terminal repeats and target site duplications, respectively. *Solid boxes* and *lines* are exons and introns of three genes marked with different colors. The *pentagons* are the last exons of the genes and indicate transcription orientation. *I*, *II*, and *III* indicate the fragments from three host genes. The cDNA sequence for each gene model is shown in parenthesis

Transposon-based gene capture is an important mechanism for gene evolution [77, 78]. Only one TRIM-mediated gene acquisition event has been reported to date, in *A. thaliana* [14]. To assess the incidence of TRIM-based gene capture, the 289 TRIM subfamilies were used for BLASTN and BLASTX searches to detect significant alignments (E value <1 × 10^−10^) to expressed genes. From this, 30 TRIM elements from seven subfamilies contained putative gene fragments, including one in *Medicago truncatula* and six in *G. max* (Additional file 1: Table S12). The sizes of the TRIMs ranged from 1,172 to 1,449 bp, similar to PACK-MULEs in rice (~1.5 kb) [37], and their internal regions had more than 70 % sequence identity to the host genes. These TRIMs contained only transcribed exon fragments, no introns. Two TRIMs carried exons from more than two genes. For instance, the internal region of GmaRetroS15 contained 217-bp and 160-bp sequences highly identical to the 5′UTR of LOC10081263 and an exon of LOC100820519, respectively. It also carried a 346-bp fragment with 76 % sequence identity to the 5–9th exons, but no introns, of LOC100798768, annotated as casein kinase I isoform delta-like protein (Fig. 3). These data suggest that TRIM-mediated gene acquisition may differ from DNA transposons, such as PACK-MULEs, that contain both exons and introns of cellular genes [78, 79], and is more similar to an LTR retrotransposon, for example, Bs1 in maize, which captured exons only [80–82], and the non-LTR retrotransposon L1 in human [83].

Among the 30 elements carrying gene fragments, all had two or more copies except GmaRetroS1 and GmaRetroS28 (Additional file 1: Table S12), all the elements contained both LTRs, and were flanked by 5-bp TSDs. One complete copy each was found for GmaRetroS1 and GmaRetroS28 in *G. max,* although other nearly complete copies were also found. This suggests that additional transposition events occurred after gene acquisition, resulting in increased copy numbers.

### Epigenetic pathways of TRIM elements

#### Methylation and targeting of TRIMs by sRNAs

**Fig. 4:**
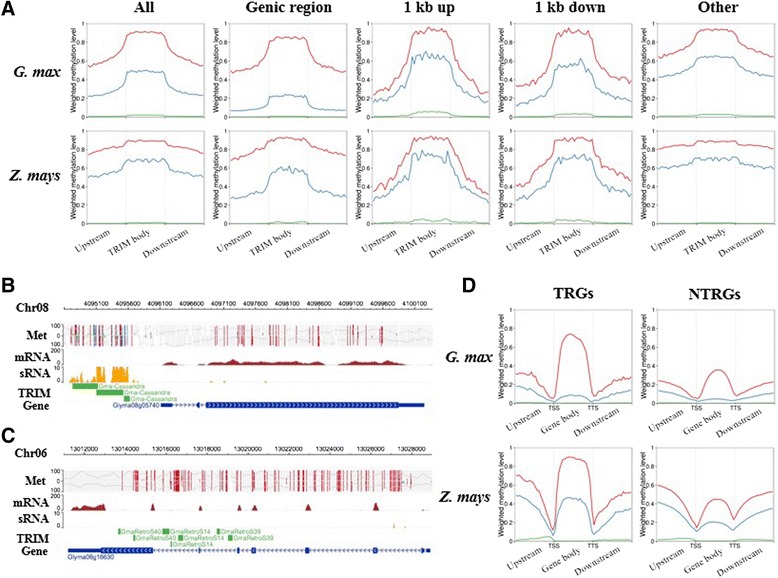
Epigenetic analyses of terminal repeat retrotransposons in miniature (*TRIMs*) in *Glycine max* and *Zea mays*. **a** Methylation patterns of TRIMs based on insertion position. *Red*: CG methylation, *blue*: CHG methylation, *green*: CHH methylation. **b** Example of TRIMs that were highly methylated and targeted by 24-nucleotide small interfering RNA. **c** Example of TRIMs that were highly methylated at only the CG context and not targeted by 24-nucleotidet small interfering RNA. **d** Methylation patterns of TRIM-related genes (*TRGs*) and non-TRIM related genes (*NTRGs*). *TSS* transcription start site, *TTS* transcription termination site

Plants have evolved multiple pathways to epigenetically regulate TEs, including DNA methylation, posttranslational histone modification, and sRNA-mediated gene silencing [84, 85]. We investigated methylation patterns and sRNA abundance of TRIMs in *G. max* and *Z. mays*. We found that TRIMs in both genomes were methylated in all three cytosine contexts (CG, CHG, and CHH, where H is A, C, or T) (Fig. 4a), and that overall methylation patterns of TRIMs were similar to those of Ty1 and Ty3 LTR retrotransposons in *G. max*. In contrast in *Z. mays*, no boundaries were found for TE bodies and flanking regions (Additional file 1: Figure S4), likely due to the extremely high TE content (85 %) in *Z. mays* [7] and the nested organization of retrotransposons, in which many LTR retroelements are inserted into other LTR retrotransposons [8]. However, the methylation patterns of TRIMs were distinct from MITEs in both *G. max* and *Z. mays* (Additional file 1: Figure S4). TRIM body methylation was similar between the two genomes but the flanking regions in *Z. mays* showed higher methylation levels than those of *G. max*. Because TRIMs were enriched in genic regions (Additional file 1: Table S4), we further investigated the methylation of TRIMs in genes, and adjacent (within 1 kb) to genes and other non-genic regions. TRIMs in genes were generally less methylated in non-CG contexts as compared to those in intergenic regions (Fig. 4a).

Methylation marks on TEs in plants are maintained by DNA methyltransferases and the RNA-directed DNA methylation pathway guided by 24-nucleotide small interfering RNAs (24 nt siRNAs) [86, 87]. To calculate the abundance of sRNA targeting TRIMs, sRNA data from *G. max* [88] and *Z. mays* [89] were mapped to the respective genomes and most TRIMs were targeted by 24 nt and/or 21 nt sRNAs (e.g., Fig. 4b, Additional file 1: Table S7). However, we also found some TRIMs located in expressed genes that were not targeted by sRNAs (Fig. 4c). Moreover, sRNA abundance varied among the different TRIM families (Additional file 1: Table S7). TRIM families were classified into three types based on DNA methylation and sRNA profiles (Additional file 1: Figure S5, Table S13): Type I: abundant 24 nt siRNAs in TE body, methylation in TE body, and relatively lower methylation in the flanking regions as compared to the TE body, showing clear borders of TRIMs; Type II: low 24 nt siRNA abundance, and CG and CHG methylation in both TE and flanking regions without clear borders; and Type III: low 24 nt siRNA abundance and high methylation only in CG context without clear borders. Thus, five, eight, and four TRIM families in *G. max* were divided into Type I, II, and III, respectively. Among six TRIM families in *Z. mays*, three were grouped into Type I and three into type II; Type III was not found in *Z. mays*. Families with high CHH methylation (Type I) were more frequently targeted by 24 nt siRNAs—the correlation between CHH methylation and sRNAs was previously reported for both *G. max* and *Z. mays* [89, 90].

#### Higher CG body methylation in TRIM-related genes

We further compared methylation levels between TRGs and NTRGs. In both *G. max* and *Z. mays*, TRGs were more methylated than NTRGs (Fig. 4d). To gain better insight into gene methylation as related to TRIM insertions, genes were categorized into three groups: (1) CG body-methylated genes, (2) C-methylated genes (possible RNA-directed DNA methylation—target loci or heterochromatic marks), and (3) unmethylated genes (Additional file 1: Table S14). TRGs had a significantly higher proportion of C methylated genes (27.4 % in *G. max* and 64.3 % in *Z. mays*) as compared to NTRGs (11.0 % in *G. max* and 35.2 % in *Z. mays*; *p*-value < 2.2 × 10^−16^, two-sample test of proportion using “prop.test” function in R). This was expected given that TRIMs were methylated in all three contexts (Fig. 4a). Interestingly, TRGs also had a significantly higher proportion of CG body-methylated genes (48.5 % in *G. max* and 19.5 % in *Z. may*) compared to NTRGs (19.8 % in *G. max* and 9.1 % in *Z. mays*; *p*-value < 2.2 × 10^−16^, two-sample test of proportion).

The proportion of CG body-methylated and C-methylated genes within TRGs varied among TRIM families (Additional file 1: Table S15). TRIM families with a higher proportion of CG body-methylated genes also had higher proportions of TRIMs inserted into genic regions, with positive correlations in both *G. max* (*R* = 0.937) and *Z. mays* (*R* = 0.438). In addition, negative correlations (*G. max*, *R* = −0.898; *Z. mays*, *R* = −0.329) were found between the proportion of C-methylated genes and rates of TRIM insertion into genic regions.

### Origin and activity of TRIMs

#### Putative autonomous retrotransposons of TRIMs

TRIMs are small elements with no coding capacity and are non-autonomous, thus mobilization depends on transposases encoded by other autonomous transposons. However, no autonomous transposon for any TRIM has been reported in plants or the red harvester ant. To identify potential autonomous elements, all 289 TRIM subfamilies were used as queries to search against the 48 plant genomes and GenBank to find related but longer elements. For most subfamilies, 278, no retrotransposase-encoding element was found, but for 11 subfamilies we identified larger, complete elements ranging in size from 3,367 to 8,504 bp, encoding proteins of 384–1,577 amino acids in length (Additional file 1: Table S16). The retroelements could be classified as either Ty1-copia or Ty3-gypsy LTR retrotransposons based on sequence similarity to other retrotransposons. The LTRs of the large retroelements exhibited 79–98 % sequence identity with the related TRIMs and the LTR sizes of the TRIMs and their larger retrotransposons were similar (Additional file 1: Table S16).

**Fig. 5:**
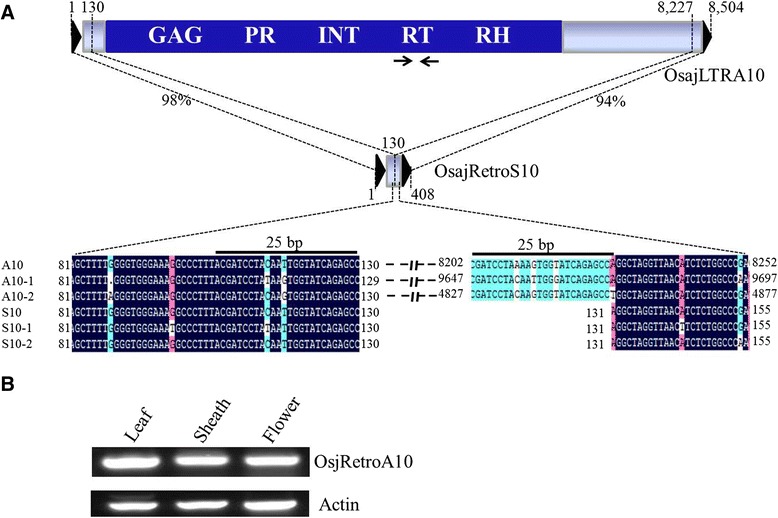
**a** OsajRetroS10 and a putative autonomous LTR retrotransposon. OsajRetroS10 is 408 bp and shares high sequence identity with 8,504-bp Ty1-copia retrotransposon OsajLTRA10 in both the LTR and internalregions. OsajLTRA10 contains a duplicated 25-bp sequence, indicated by *black lines*. Primers targeting the conserved domain of the reverse transcriptase (RT) are indicated by *arrows*. **b** RT-PCR analysis of OsajLTRA10

Sequence similarity between the large elements and the TRIMs was not restricted to LTR regions. We identified an 8,504-bp Ty1-copia retrotransposon, OsajLTRA10, in Nipponbare (*Oryza sativa* L. ssp. *japonica*) using the 408-bp TRIM OsajRetroS10 as a query. The LTRs of both elements were 115 bp and shared 97 % sequence identity. OsajRetroS10 also showed 98 % and 94 % sequence identity with OsajLTRA10 at positions 1–130 and 131–408, respectively, which covers all of OsajRetroS10 (Fig. 5a). From this, we deduced that OsajRetroS10 is a derivative of OsajRetroA10 via internal deletions, with a breakpoint near the 130th nucleotide of OsajLTRA10. There were three complete OsajLTRA10 elements in Nipponbare, including OsajLTRA10 on chromosome 1 and two other copies [OsajLTRA10-1 (9,948 bp, on chromosome 9) and OsajLTRA10-2 (5,124 bp, on chromosome 12)]. Sequence alignment of OsajLTRA10 elements and OsajRetroS10 TRIMs revealed that the complete elements contained a 25-bp sequence (CGATCCTA(C/T)AA(G/T)TGGTATCAGAGCC) immediately 5′ of the breakpoint site, and the three OsajLTRA10 elements contained another nearly identical 25-bp sequence immediately 3′ of the breakpoint site. We refer to this as the “duplicated internal sequence.” The 25-bp duplicated internal sequence were also found in OsaiLTRA10 in 93–11 (*Oryza sativa* L. ssp. *indica*), a close relative of Nipponbare.

Among the 11 large LTR retrotransposons, SlyLTRA4, PtrLTRA2, VviLTRA5, PbrLTRA6, CarLTRA1, CarLTRA2, and GmaLTRA2 are likely unable to mobilize TRIMs because their retrotransposon proteins are either short or truncated. The remaining four elements encode retrotransposases that contain all functional domains for retrotransposition: SitLTRA5 has a 1,409 amino acid sequence, OsajLTRA10 a 1,577 amino acid sequence, OsiLTRA10 a 1,431 amino acid sequence, and SmoLTRA4 a 1,218 amino acid sequence. Thus, these four LTR retrotransposons are putative autonomous elements that can mobilize their related TRIMs. Furthermore, multiple expressed sequence tags (ESTs) showing sequence similarity with these four retrotransposons were identified, confirming the transcriptional activity of these LTR retrotransposons. We performed reverse transcriptase (RT) PCR analysis to validate the expression of OsajLTRA10 using primers complementary to the RT domain (Fig. 5a). Significant amplification was detected using cDNA from leaf, sheath, and flower of Nipponbare and confirmed the transcriptional activity of the OsajLTRA10 transposon (Fig. 5b).

#### Recent transpositions of a TRIM family

To gain more insight into the activity of TRIMs, we compared TRIMs from the reference genomes for two rice subspecies, *japonica* and *indica*, that diverged ~0.2–0.4 Mya from either *O. nivara* or *O. rufipogon* [91], and identified 41 and 31 polymorphic TRIMs in Nipponbare and 93–11, respectively. All polymorphic elements were flanked by 5-bp TSDs and absent in the orthologous regions. This suggests that these are newly inserted TRIMs and that transposition of TRIMs may be similar to that of LTR retrotransposons, as both create 5-bp TSDs.

We next conducted PCR to validate the new insertions of OsaRetroS10, for which a putative autonomous retrotransposon was found in both Nipponbare and 93–11 (Fig. 5a, Additional file 1: Table S16). We used three pairs of primers targeted to the flanking regions of new insertion sites (Additional file 1: Figure S6A) to amplify DNA from seven rice varieties, including four *japonica* (Nipponbare, Kitaaki, Azucena, and Moroberkan), three *indica* (93–11, IR36, and IR64), and two AA wild relatives, *O. nivara* and *O. rufipogon*. All three primer pairs yielded expected PCR product sizes in both Nipponbare and 93–11 and the two wild rice species (Additional file 1: Figure S6B), indicating that these TRIMs were mobilized after the divergence of these two rice subspecies. Interestingly, smaller bands were found in Kitaaki with P1 primers and IR64 with P2 primers. Sequence analysis did not show a deletion in either Kitaaki or IR64, rather an extra complete element and 5-bp sequence were found in the insertion site of Nipponbare and 93–11, respectively. This indicates that OsaRetroS10 may still be active in rice.

## Discussion

### Detection and comparison of TRIMs across the plant kingdom

Owing mostly to their diminutive sizes and lack of conserved coding sequences, TRIMs have been difficult to annotate. The first TRIM was identified during analysis of the urease gene using dot plot software [14]. Since then, other TRIMs have been discovered through comparison of orthologous sequences [16] or by PCR cloning experiments [17, 92]. However, these approaches are time consuming and not suited for genome-wide identification of TRIMs. Computational tools have been developed for de novo identification and classification of LTR retrotransposons (e.g., LTR_STRUC [93], LTR_FINDER [66], LTRharvest [94], and LTRdigest [95]). However, these tools have limited application for finding TRIMs. For instance, the LTR_STRUC program is inefficient at detecting small retrotransposons (less than 1,000 bp); thus, the majority of TRIMs would be missed. Both LTR_FINDER and LTRharvest allow users to define search parameters to find short elements, but will miss diverged elements that lack the primer binding site and/or polypurine tract. LTRdigest requires a retrotransposase sequence, lacking in TRIMs.

In this study, we combined de novo annotation and homology-based searches to annotate plant TRIMs in 48 genomes. This combined approach detected more TRIMs than simply using de novo annotation and provides a strategy to identify TRIMs in other genomes. For example, of the 11 TRIM subfamilies in *S pimpinellifolium*, no TRIM was detected by LTR_FINDER, and all these were found by homology-searches using TRIMs from *S. lycopersicum* and others. Furthermore, current annotation tools are not suited for short DNA sequences such as ESTs and genome survey sequences, whereas homology-based searches can detect TRIMs in these datasets. Although TRIMs have not been reported in animals, excepting the red harvester ant, this method also works for identifying TRIMs in animals where TRIMs may have been missed by traditional transposon annotations, given that we found new TRIMs in human, mouse, and nematode (Gao et al. unpublished data).

Most comparisons of TEs have been limited to closely related species [11, 96] or performed at the protein level with conserved transposase domains [97]. This is because transposons from distantly related plants are often diverged at the nucleotide level; thus, it is difficult to compare and classify transposons from distantly related genomes, particularly for fragmented elements and those that lack transposon proteins.

TRIMs are unusual elements that have been mostly ignored during the annotation of plant genomes—only 11 TRIM families had been reported in flowering plants thus far. In this study, we used 48 genomes that span ~610 My of plant evolutionary history [98] to identify TRIMs in flowering plants, lycophytes, and algae. TRIMs from these species were grouped into 156 TRIM families including 145 new families. Of these families, 104 were shared across a range of taxonomic groups. To our knowledge, this is the most comprehensive exploration and classification of TRIMs in the plant kingdom. These results provide a valuable resource to the genomics community for identification of homologous TRIM elements in newly sequenced genomes.

### Origin and transpositions of TRIMs

No autonomous element has been reported for any of the previously reported TRIMs [14–17, 19]. Thus, the evolutionary origin and transposition mechanism of TRIMs remains ambiguous. We found 11 large LTR retrotransposons that share high sequence similarity with specific TRIM LTRs and internal regions and have similarly sized LTRs (Additional file 1: Table S16, Fig. 5). This is the first direct evidence that TRIMs may be derived from LTR retrotransposons. Notably, the large retroelements identified from the six flowering plants were all Ty1-copia types, whereas the large retrotransposon from *S. moellendorffii* was a Ty3-gypsy type. It is tempting to speculate that this may reflect an origin for TRIMs from Ty1-copia elements in flowering plants versus Ty3-gypsy elements in *S. moellendorffii*; however, additional genome sequences are needed to test this hypothesis.

Of the 11 TRIM-related LTR retrotransposons (Additional file 1: Table S16), seven encode short or truncated proteins and are likely non-autonomous LTR retrotransposons. However, four encode full retrotransposases and are putative autonomous elements for TRIMs. Our genome-wide comparisons of TRIMs between two subspecies of *O. sativa* and subsequent PCR survey confirmed recent transpositional activity of OsajRetroS10 in *O. sativa*, which contains a related, autonomous LTR retrotransposon.

Of the identified 289 TRIM subfamilies, only 11 have related larger LTR retrotransposons. Some full retrotransposons may have been missed owing to incomplete genome assemblies. Alternatively, this may reflect selective pressures in plant genomes where transposons are subjected to strong selective pressure to avoid disruption of host genes [99]. However, many TRIMs are highly conserved across species and have likely colonized plants for more than ten million years (Table 1, Fig. 1), though we cannot completely exclude the possibility of horizontal transfer. This leads to questions of how and why TRIMs are retained over such long evolutionary times and not removed via mutation or deletion? One strategy may be that TRIMs are small and often insert into noncoding regions, such as introns, and have no effect on gene function and host fitness and are generally neutral, similar to MITEs [73].

### Unique and evolutionary features of TRIMs

Even though TRIMs are similar in structure to LTR retrotransposons, there are several differentiating features. First and most obvious is their diminutive size. We found that the sizes of more than 77 % of the identified TRIMs were less than 1,000 bp, much smaller than most LTR retrotransposons. Therefore, unlike LTR retrotransposons, the amplification of TRIMs has had less impact on genome expansion. Notably, the smallest TRIM, CcaRetroS9 in *C. cajan*, was only 233 bp, with 52-bp LTRs with 10 complete copies in the genome.

Second, TRIMs are enriched in or near genic regions. Even though LTR retrotransposons contribute large fractions of plant genomes, most are concentrated in highly heterochromatic regions [9–12]. For example, these elements account for 75 % of the maize genome [7] but only ~10 % are found in or near genes (Additional file 1: Table S5). Our results show that TRIMs are more frequently inserted or retained in genic regions (Additional file 1: Table S4), at a significantly higher frequency than both Ty1 and Ty3 LTR retrotransposons (Additional file 1: Table S5, S9). We also observed that TRGs are larger than those without TRIMs (Additional file 1: Table S6, S10), which may reflect a preference for insertion or retention in larger genes because homologs of TRGs were also large (Additional file 1: Table S8). Previous studies revealed a negative association between gene expression and gene length. That is, smaller genes are usually highly expressed and larger ones are more moderately transcribed [100, 101]. However, larger genes are more likely to have alternative splicing and other genomic novelties due in part to the insertion of TEs [102]. Indeed, we identified 12 TRIM-related and expressed genes present only in *G. max* and 32 in *Z. mays*, which may represent new genes, or genes for which the homologous genes were either absent or highly diverged in other species. This included genes in which TRIM insertions led to changes in gene structure (Additional file 1: Figure S6).

Third, TRIMs are conserved in plant genomes over long evolutionary timeframes. LTR retrotransposons are dynamic and rapidly diverging sequences [9, 11, 96], with few exceptions (e.g., centromeric retrotransposons that are shared within the grass family [10]). Most plant LTR retroelements are present in only a single genome or in closely related genomes. In contrast, 104 (67 %) TRIM families were shared within plant families and/or between distantly related species (Fig. 1, Table 1), which may indicate that TRIMs are conserved in plants even though we cannot completely exclude the possibility of their horizontal transfer. Through comparative analyses, we found 55 TRIMs located in the orthologous regions of *G. max* and *P. vulgaris*, and five were shared across three species, *G. max*, *C. cajan*, and *P. vulgaris* (Additional file 1: Figure S8). Thus, TRIMs are able to colonize and be retained in plants over a longer evolutionary period than typical LTR retrotransposons [103]. There are a few potential reasons for this unusual conservation: (1) TRIMs are small so there is less opportunity for nested insertions or truncations leading to degradation; and (2) elements in genic and non-genic regions evolve differently, and because TRIMs were often found in or near genes, they have likely undergone stronger purifying selection [104].

Fourth, TRIMs were associated with CG body-methylated genes. The characteristics of TRGs (longer gene length, higher number of exons, and lower evolutionary rate) are similar to the characteristics of CG body-methylated genes [105–107]. Moreover, significantly higher proportions of TRGs were found in CG body-methylated genes in *G. max* and *Z. mays* (Additional file 1: Table S14). This suggests that TRIMs either more frequently insert into or are retained in CG body-methylated genes. Interestingly, different TRIM families exhibited distinct methylation patterns and TRIM families with higher insertion frequencies into genic regions were more likely to be in CG body-methylated genes (Additional file 1: Table S15). Given that CG body-methylated genes show lower evolutionary rates [106, 107] and are moderately expressed as compared to unmethylated genes [106, 108], CG body-methylated genes could be under strong purifying selection to retain these genes. If the insertion of a TRIM does not interfere with the function and/or expression of the host gene, the TRIM could survive and be retained longer along with the CG body-methylated genes. This may be one reason why TRIMs that were found in CG body-methylated genes were either not methylated or showed only high CG methylation, unlike other TEs that were highly methylated in all three contexts. Alternatively, the insertion of methylated TRIMs in all three contexts (e.g., Fig. 4a) could alter the methylation and expression of the host gene and are therefore removed under purifying selection, resulting in the low rate of the genic insertions, as seen for other TEs. Taken together, TRIMs incorporated into CG body-methylated genes tend to survive over long evolutionary periods, in contrast to other TEs.

Finally, TRIMs are a distinct transposon group from MITEs based on the following: (1) TRIMs are structurally similar to LTR retrotransposons and have direct terminal repeats, whereas MITEs are similar to DNA transposons with terminal inverted repeats; (2) compared to MITEs, we found TRIMs in larger genes with more exons (Additional file 1: Table S10), though it is not clear if this reflects an insertional bias or some selective pressure on MITEs and TRIMs; (3) the overall methylation patterns of TRIMs are similar to LTR retrotransposons but distinct from MITEs (Additional file 1: Figure S4); and (4) TRIMs are derived from internal deletions of LTR retrotransposons, whereas MITEs are generated by DNA transposons and move via a cut-and-paste model [109]. Even though they are grouped into different classes of transposons, TRIMs and MITEs do have some commonalities, such as small sizes and preferential insertion or maintenance in genic regions, especially in introns.

## Conclusions

We conducted the most comprehensive analysis of TRIMs thus far and found that these elements were distributed and conserved across a range of plant species and could be tandemly arrayed. Our results also suggested that TRIMs appear to be derived from LTR retrotransposons and, in a few species, autonomous LTR retrotransposons were found that likely mobilize TRIMs, although the interactions between TRIMs and the potential autonomous retrotransposons needs to be verified by additional experiments. TRIMs were frequently enriched in larger genes and have contributed to genetic novelty, including UTRs, exons, and the creation of new genes. TRGs have undergone strong purifying selection and were highly methylated in the CG context. Thus, from an evolutionary and functional perspective, TRIMs are potentially important sources of genetic novelty but have received scant attention during genome annotation and analysis. Our data provide a holistic view of TRIMs and their unique roles in the plant kingdom, and expands our understanding of plant genome evolution as mediated by LTR retrotransposons.

## Methods

### Plant materials

A total of 10 plant genotypes were used in this study, including the inbred line B73 used for the maize genome sequencing project; two wild rice species, *O. nivara* and *O. rufipogon*; and seven cultivated rice species, Nipponbare, Kitaaki, Azucena, Moroberkan, 93–11, IR36, and IR64. The seeds of all these plants were planted and grown in the greenhouse at the University of Georgia with the temperatures set at 30 °C/25 °C (day/night) and a photoperiod at 12 h light/12 h dark. DNAs were extracted from leaves using a cetyltrimethylammonium bromide method.

### Plant genome sequences and datasets

We used 48 whole genome sequences from a wide evolutionary range of plants for annotation of TRIMs. The information for these genomes, gene annotation, and availability are shown in Additional file 1: Table S1. Only the genomes published as of 1 April 2013 were included. Additionally, the transposon database for *G. max* and *Z. mays* were downloaded from the maize transposable element (TE) database (http://maizetedb.org/~maize) and the USDA-ARS soybean genetics and genomics database (http://www.soybase.org/search).

### TRIM annotation and classification

We combined de novo annotation and homology-based searches to discover TRIM elements. First, the 48 genomes were analyzed using LTR_FINDER [66] with default parameters, except that we set a 30-bp minimum and 500-bp maximum LTR length, and 30-bp minimum and 2,000-bp maximum distance between 5′ and 3′ LTRs. The output sequences of all TRIMs were then manually inspected to discard incorrectly predicted sequences and to determine the exact boundaries of TRIMs. Additionally, all TRIM sequences were used as queries to conduct BLASTX searches against the identified proteins of retrotransposons to exclude sequences that contained retrotransposases (E value < 10^−5^). We used three criteria to define a TRIM element: (1) the element size should be less than 1,500 bp and without gaps; (2) there shoud be at least two complete copies or one complete element and one solo LTR, and each of the copies should be flanked by different TSDs; and (3) the element should not contain retrotransposon proteins.

Second, all de novo annotated TRIM sequences from each genome were grouped into subfamilies following a previous publication [110]; elements sharing at least 80 % identity over 80 % of the element length were grouped together. We used “subfamily” to define TRIMs in each of the plant genomes because TRIM elements are conserved between related species and homologous elements from same TRIM family may be present in different genomes [14, 15, 17],

Third, a representative element for each TRIM subfamily annotated by LTR_FINDER and the previously reported TRIMs in plants [14–17] were combined for BLASTN searches against each of the 48 plant genomes and GenBank to detect significant hits (E value < 10^−5^) using different options, including nucleotide collection (nr/nt), reference genomic sequences, ESTs, genomic survey sequences, high throughput genomic sequences, and whole-genome shotgun contigs. The aims of these searchers were to identify TRIMs missed by LTR_FINDER and determine if each of the TRIM elements was conserved or species specific.

Finally, the TRIMs annotated by LTR_FINDER and homology searches were combined to conduct all-against-all BLASTN searches to group all TRIMs into families, using the criteria that TRIM elements from different genomes show significant sequence similarity (E value < 1 × 10^−5^) over 50 bp and 5 % of the complete element size. These criteria were used to determine if TRIMs were species specific. If no significant hit was found outside the host genome (either the other 47 species or GenBank), the element was considered species specific.

To estimate the copy number and abundance of TRIMs, TRIM elements were used as a custom library to screen the plant genomes with RepeatMasker (http://www.repeatmasker.org) using default parameters with the “nolow” option. We also set a cutoff score greater than 250 and hit sequence size longer than 50 bp.

### Identification of TRIM-related genes and homologs and definition of syntenic blocks

A custom perl script was used to screen the Repeat Masker output files from 14 plant genomes (Additional file 1: Table S4) against GFF3 annotation files downloaded from Phytozome (http://phytozome.jgi.doe.gov/pz/portal.html) and to identify TRGs by comparing the positions of TRIMs and annotated genes in the genomes. To avoid duplicated counting, TRIMs that spanned both exon and intron or upstream and exon were considered a single exon. To find the homologous genes in the relative species, all proteins of annotated genes from *G. max* and *Z. mays* were extracted and used as queries to conduct BLASTP searches against the protein sequences of the annotated genes in four related genomes, *C. cajan*, *P. vulgaris*, *S. bicolor*, and *O. sativa*. The proteins that showed significant sequence similarity (E value < 1 × e^−10^) with the query proteins were considered homologous genes. If multiply significant hits were detected for a same gene, only the sequence with the lowest E value was considered. The syntenic genes shared by *G. max/P. vulgaris* and *Z. mays/S. bicolor* were obtained from the Plant Genome Duplication Database website; all these syntenic blocks were defined by combining BLASTP searches and package computational programs [74, 75].

### PCR and RT-PCR analysis

We performed PCR and RT-PCR analysis following previous protocols [15]. Briefly, the DNAs from cultivated and wild rice and maize were amplified with the corresponding primers (Additional file 1: Table S16) to validate insertion polymorphisms of a TRIM in rice and TA-TRIMs in maize, respectively. All amplification reactions were done using an MJ Research PTC-200 thermal cycler and the PCR products were purified with QIAquick PCR purification kits (QIAGEN, Venlo, Netherlands) and sequenced by GENEWIZ, Inc. (South Plainfield, NJ, USA). To detect the transcription activity of the rice retrotransposon OsajLTRA10, we collected the leaves and sheath of 4-week-old plants and 2–3 cm young spikes from Nipponbare. Total RNA was isolated using the TRIZOL Reagent (Invitrogen, Carlsbad, CA, USA). Four micrograms total RNA from each sample was converted into single-strand cDNA with reverse transcriptase (Invitrogen). The cDNA reactions were then diluted 4–5-fold, and 2 μL of the diluted cDNA was used as templates for PCR amplifications with the primers targeted to the retrotransposon and actin gene (Additional file 1: Table S17).

### Calculation of evolutionary rates

The genome-wide non-synonymous substitution (*Ka*) and synonymous substitution (*Ks*) rates were calculated using the gKaKs computational pipeline [76] with the default parameters. Briefly, the annotated genes in *G. max* and *Z. mays* were used as queries to search against *P. vulgaris* and *S. bicolor*, respectively, using BLAT [111]. The orthologous gene pairs were aligned via bl2seq [112], and *Ka* and *Ks* for each homologous sequence pair was calculated using codeml from the PAML package [113].

### Methylation and sRNA analysis of TRIM

The methylome data of soybean (GenBank accession L: PRJNA264602) [105] and maize (GenBank accession: GSE39232) [89] were used to determine the methylation profiles of TRIM. The mapping and calling of methylation were done as described [89, 105] with modifications. Briefly, raw reads containing low quality (<Q30) or primer/adaptor sequences were trimmed using Cutadapt [114]. Trimmed reads were aligned to either the soybean [38] or maize genome [7] using Bismark v0.13.1 [115] and only uniquely mapped reads were retained. To reduce potential biases in calling methylation, clonal reads generated from PCR amplification were removed and sequence bases showing extreme methylation levels were excluded from further analysis. Methylated cytosines were determined using the binomial distribution as described by Lister et al. [116]. The bisulfite non-conversion rates were estimated from the percentage of cytosine bases sequenced at reference cytosine positions in the chloroplast or unmethylated Lambda genomes. The methylation profiles of TRIM were determined as weighted methylation levels [117].

The published sRNA data of soybean [88] and maize [89] were used to determine the abundance of sRNA targeting TRIM. Adapter and quality-trimmed reads matching transfer RNAs, ribosomal RNAs, small nuclear RNAs, and small nucleolar RNAs were excluded. Filtered reads were mapped against either soybean [38] or maize [7] using Bowtie2 [118], accepting only perfect matches. Mapped reads were normalized to transcripts per million using HTSeq [119] to account for varying sequencing depth.

## Data availability

All TRIM sequences identified by this and previous studies can be accessed via http://bit.ly/1Rtqkie. The plant genome sequences are available from the National Center for Biotechnology Information (NCBI) and additional websites. (Reference sequence URLs and accession numbers can be found in Additional file 1: Table S1).

## Ethics approval

Ethics approval was not needed for this study.

## Additional file

Additional file 1:
**Tables S1 to S17, Figs S1 to S8.** (DOCX 1296 kb)

## Abbreviations

bp: base pair
EST: expressed sequence tag
Ka: non-synonymous substitutions per non-synonymous site
kb: kilobase
Ks: synonymous substitutions per synonymous site
LTR: long terminal repeat
MITEs: miniature inverted-repeat transposable elements
My: million years
Mya: million years ago
NTRGs: non-terminal repeat retrotransposon in miniature-related genes
RT-PCR: reverse transcription polymerase chain reaction
TA-TRIM: tandemly arrayed terminal repeat retrotransposon in miniature
TE: transposable element
TRGs: terminal repeat retrotransposon in miniature-related genes
TRIM: terminal repeat retrotransposon in miniature
TSD: target site duplication
UTR: untranslated regions
