# Supplementary material for: Landscape and evolutionary dynamics of terminal repeat retrotransposons in miniature in plant genomes
Source: Genome Biol. 2016 Jan 18;17:7. doi: 10.1186/s13059-015-0867-y (PMC4717578; doi:10.1186/s13059-015-0867-y)
Supplement: Additional file 1: — Tables S1 to S17, Figs S1 to S8. (DOCX 1296 kb) [file 13059_2015_867_MOESM1_ESM.docx]

**Supplemental Material for**

**Landscape and evolutionary dynamics of terminal-repeat retrotransposons in miniature (TRIMs) in 48 whole plant genomes**

Dongying Gao^1^, Yupeng Li^1^, Kyung Do Kim^1^, [Brian](http://www.caes.uga.edu/applications/personnel/profile.cfm?ID=13969) Abernathy^1^, Scott A. Jackson^1*^

^1^Center for Applied Genetic Technologies, University of Georgia, 111 Riverbend Rd., Athens, GA 30602. USA

*Corresponding author:

Scott A. Jackson

E-mail: [sjackson@uga.edu](mailto:sjackson@uga.edu)

**This file includes**

Supplemental Tables S1 to S17

Supplemental Figs. S1 to S8

**Supplemental Table S1. List of 48 sequenced plant genomes used in this study**

| Genomes/Division | Website-Version (GenBank ID) | Reference |
| --- | --- | --- |
| *Solanum lycopersicum* (Tomato) /[Eudicot](http://en.wikipedia.org/wiki/Eudicots) | http://solgenomics.net  (CM001064- CM001075) | Tomato Genome Consortium (2012) |
| *Solanum pimpinellifolium* (Currant Tomato) /[Eudicot](http://en.wikipedia.org/wiki/Eudicots) | [http://solgenomics.net](https://bl2prd0210.outlook.com/owa/redir.aspx?C=gn6g5kBYZ0uX8bD6cldpe-bQVAXIIc8I0Wmm_HUf9s7b3TYwpa_m-eSMYD9cEKNSMerP6B-tVHs.&URL=http%3a%2f%2fsolgenomics.net%2forganism%2fSolanum_pimpinellifolium%2fgenome)  (AGFK01000001-AGFK01309180) | Tomato Genome Consortium (2012) |
| *Solanum tuberosum* L (Potato) /[Eudicot](http://en.wikipedia.org/wiki/Eudicots) | <http://www.potatogenome.net>-PGSC_DM_v3_2.1.10 (JH137791-JH152643) | Xu et al (2011) |
| *Cucumis sativus* (Cucumber) /[Eudicot](http://en.wikipedia.org/wiki/Eudicots) | http://www.phytozome.net-Csativus_122 (GL376737-GL377301) | Huang et al (2009) |
| *Cucumis melo* (Melon) /[Eudicot](http://en.wikipedia.org/wiki/Eudicots) | https://melonomics.net-V3.5  (HF534877-HF536475) | Garci-Mas Jet al (2012) |
| *Citrullus lanatus* (Watermelon) /[Eudicot](http://en.wikipedia.org/wiki/Eudicots) | <http://www.icugi.org-V>1  (AGCB01000001-AGCB01040248) | Guo et al (2013) |
| *Malus x domestica* (Apple) /[Eudicot](http://en.wikipedia.org/wiki/Eudicots) | http://www.phytozome.net-Mdomestica_196 (CM001026-CM001042) | Velasco et al (2010) |
| *Prunus mume* (Plum blossom) /[Eudicot](http://en.wikipedia.org/wiki/Eudicots) | http://prunusmumegenome.bjfu.edu.cn (CM001826-CM001833) | Zhang et al (2012) |
| *Pyrus bretschneideri* (Pear) /[Eudicot](http://en.wikipedia.org/wiki/Eudicots) | [http://peargenome.njau.edu.cn](http://peargenome.njau.edu.cn/)  ([AJSU01000001-AJSU01026566](http://www.ncbi.nlm.nih.gov/Traces/wgs?val=AJSU01#contigs)) | Wu et al (2013) |
| *Fragaria vesca* (Woodland strawberry) /[Eudicot](http://en.wikipedia.org/wiki/Eudicots) | [http://www.rosaceae.org](https://bl2prd0210.outlook.com/owa/redir.aspx?C=gn6g5kBYZ0uX8bD6cldpe-bQVAXIIc8I0Wmm_HUf9s7b3TYwpa_m-eSMYD9cEKNSMerP6B-tVHs.&URL=http%3a%2f%2fwww.rosaceae.org%2fprojects%2fstrawberry_genome)-fvesca_v1.1  (CM001053-CM001059) | Shulaev et al (2011) |
| *Cannabis sativa* (Marijuana) /[Eudicot](http://en.wikipedia.org/wiki/Eudicots) | http://www.ncbi.nlm.nih.gov  (AGQN01000001-AGQN01337115) | [van Bakel et](http://www.ncbi.nlm.nih.gov/pubmed?term=van%20Bakel%20H%5BAuthor%5D&cauthor=true&cauthor_uid=22014239) al (2011) |
| *Lotus japonicus* (Lotus) /[Eudicot](http://en.wikipedia.org/wiki/Eudicots) | http://www.kazusa.or.jp-lotus_r2.5  (DF093176–DF093536) | Sato et al (2008) |
| *Medicago truncatula* (Barrel medic) /[Eudicot](http://en.wikipedia.org/wiki/Eudicots) | http://www.phytozome.net-Mtruncatula_135 (CM001217- CM001224) | Young et al (2011) |
| *Cicer arietinum* (Chickpea) /[Eudicot](http://en.wikipedia.org/wiki/Eudicots) | <http://www.ncbi.nlm.nih.gov>  (CM001764-CM001771) | [Varshney et](http://www.ncbi.nlm.nih.gov/pubmed?term=Varshney%20RK%5BAuthor%5D&cauthor=true&cauthor_uid=22057054) al (2013) |
| *Glycine max* (Soybean) /[Eudicot](http://en.wikipedia.org/wiki/Eudicots) | http://www.phytozome.net-Gmax_109  (CM000834-CM000853) | Schmutz et al (2010) |
| *Cajanus cajan* (Pigeonpea) /[Eudicot](http://en.wikipedia.org/wiki/Eudicots) | http://www.ncbi.nlm.nih.gov  ([AFSP01000001-AFSP01191705](http://www.ncbi.nlm.nih.gov/Traces/wgs?val=AFSP01#contigs)) | [Varshney et](http://www.ncbi.nlm.nih.gov/pubmed?term=Varshney%20RK%5BAuthor%5D&cauthor=true&cauthor_uid=22057054) al (2011) |
| *Jatropha curcas* (Sanskrit) /[Eudicot](http://en.wikipedia.org/wiki/Eudicots) | [http://www.kazusa.or.jp](https://bl2prd0210.outlook.com/owa/redir.aspx?C=gn6g5kBYZ0uX8bD6cldpe-bQVAXIIc8I0Wmm_HUf9s7b3TYwpa_m-eSMYD9cEKNSMerP6B-tVHs.&URL=http%3a%2f%2fwww.kazusa.or.jp%2fjatropha%2f)-JAT_r3.0 (BABX01000001-BABX01150417) | Sato et al (2011) |
| *Linum usitatissimum* (Flax) /[Eudicot](http://en.wikipedia.org/wiki/Eudicots) | http://www.phytozome.net-Lusitatissimum_200 (AFSQ01000001-AFSQ01048397) | [Wang](http://www.ncbi.nlm.nih.gov/pubmed?term=Wang%20Z%5BAuthor%5D&cauthor=true&cauthor_uid=22757964)  et al (2012) |
| *Ricinus communis* (Castor bean plant) /[Eudicot](http://en.wikipedia.org/wiki/Eudicots) | http://www.phytozome.net-Rcommunis_119 (EQ973772-EQ999533) | Chan et al (2010) |
| *Populus trichocarpa* (Western poplar) /[Eudicot](http://en.wikipedia.org/wiki/Eudicots) | http://www.phytozome.net- Ptrichocarpa_156 (CM000337-CM000355) | Tuskan et al (2006) |
| *Arabidopsis thaliana* (Thale cress) /[Eudicot](http://en.wikipedia.org/wiki/Eudicots) | http://www.phytozome.net-Athaliana_167 (CP002684-CP002688) | Arabidopsis Genome Initiative (2000) |
| *Arabidopsis lyrata* (Lyrate rockcress) /[Eudicot](http://en.wikipedia.org/wiki/Eudicots) | http://www.phytozome.net-Alyrata_107 (GL348713-GL349407) | Hu et al (2011) |
| *Thellungiella salsuginea =* *Eutrema salsugineum* /[Eudicot](http://en.wikipedia.org/wiki/Eudicots) | http://www.ncbi.nlm.nih.gov  (AHIU01000001-AHIU01028682) | Wu et al (2012) |
| *Brassica rapa* (Turnip mustard) /[Eudicot](http://en.wikipedia.org/wiki/Eudicots) | http://www.phytozome.net-Brapa_197  (CM001634-CM001643) | Wang et al (2011) |
| *Thellungiella parvula* (Eutrema parvulum) /[Eudicot](http://en.wikipedia.org/wiki/Eudicots) | http://www.ncbi.nlm.nih.gov  (CM001187-CM001193) | [Dassanayake](http://www.ncbi.nlm.nih.gov/pubmed?term=Dassanayake%20M%5BAuthor%5D&cauthor=true&cauthor_uid=21822265) (2011) |
| *Carica papaya* (Papaya) /[Eudicot](http://en.wikipedia.org/wiki/Eudicots) | http://www.phytozome.net-Cpapaya_113 (DS981520-DS984726) | Ming et al (2008) |
| *Theobroma cacao* (Cocoa) /[Eudicot](http://en.wikipedia.org/wiki/Eudicots) | http://www.ncbi.nlm.nih.gov  (CACC01000001-CACC01025912) | Argout et al (2011) |
| *Gossypium raimondii* (Cotton) /[Eudicot](http://en.wikipedia.org/wiki/Eudicots) | http://www.phytozome.net-Graimondii_221 (CM001740-CM001752) | Paterson et al (2012) |
| *Vitis vinifera* (Grape vine) /[Eudicot](http://en.wikipedia.org/wiki/Eudicots) | http://www.phytozome.net-Vvinifera_145 (FN594950-FN597014) | Jaillon et al (2007) |
| *Citrus sinensis* (sweet orange) /[Eudicot](http://en.wikipedia.org/wiki/Eudicots) | http://www.phytozome.net-Csinensis_154 (CM001701-CM001709) | Xu et al (2013) |
| *Sorghum bicolor* (Sorghum) /[Monocot](http://en.wikipedia.org/wiki/Eudicots) | http://www.phytozome.net-Sbicolor_79 (CM000760-CM000769) | Paterson et al (2009) |
| *Zea mays* (Maize) /[Monocot](http://en.wikipedia.org/wiki/Eudicots) | http://www.phytozome.net- Zmays_181 (NC_024459-NC_024468) | Schnable et al (2009) |
| *Setaria italica* (Foxtail millet) /[Monocot](http://en.wikipedia.org/wiki/Eudicots) | http://www.phytozome.net-Sitalica_164 (JH667841-JH668176) | Bennetzen et al (2012) |
| *Oryza sativa* (Asian cultivated rice) Indica /[Monocot](http://en.wikipedia.org/wiki/Eudicots) | <http://www.ncbi.nlm.nih.gov>  (CM000126-CM000137) | Yu et al (2002) |
| *Oryza sativa* (Asian cultivated rice) Japonica /[Monocot](http://en.wikipedia.org/wiki/Eudicots) | http://rice.plantbiology.msu.edu-V7  (AP008207-AP008218) | IRGSP (2005) |
| *Oryza brachyantha* /[Monocot](http://en.wikipedia.org/wiki/Eudicots) | http://rice.genomics.org.cn  (CM001241-CM001252) | Chen et al (2013) |
| *Brachypodium distachyon* (Purple false brome) /[Monocot](http://en.wikipedia.org/wiki/Eudicots) | http://www.phytozome.net-Bdistachyon_192 (CM000880-CM000884) | International Brachypodium Initiative (2010) |
| *Phoenix dactylifera* (Date palm) /[Monocot](http://en.wikipedia.org/wiki/Eudicots) | [http://qatar-weill.cornell.edu](https://bl2prd0210.outlook.com/owa/redir.aspx?C=gn6g5kBYZ0uX8bD6cldpe-bQVAXIIc8I0Wmm_HUf9s7b3TYwpa_m-eSMYD9cEKNSMerP6B-tVHs.&URL=http%3a%2f%2fqatar-weill.cornell.edu%2fresearch%2fdatepalmGenome%2fdownload.html)-PdactyKAsm30_r20101206  (GL739410-GL758109) | [Al-Dous](http://www.ncbi.nlm.nih.gov/pubmed?term=Al-Dous%20EK%5BAuthor%5D&cauthor=true&cauthor_uid=21623354) et al (2011) |
| *Musa acuminata* (Banana) /[Monocot](http://en.wikipedia.org/wiki/Eudicots) | http://banana-genome.cirad.fr-V1  ([CAIC01000001–CAIC01024424](http://www.ncbi.nlm.nih.gov/nuccore/?term=CAIC01000001–CAIC01024424)) | D'Hont et al (2012) |
| *Selaginella moellendorffii* (Spikemoss) /Lycophyte | http://www.phytozome.net-Smoellendorffii_91 (GL377565-GL378322) | [Banks](http://www.ncbi.nlm.nih.gov/pubmed?term=Banks%20JA%5BAuthor%5D&cauthor=true&cauthor_uid=21551031)  et al (2011) |
| *Physcomitrella patens* (Moss) /Bryophyte | http://www.phytozome.net-Ppatens_152 (DS544890-DS546995) | Rensing et al (2008) |
| *Chlamydomonas reinhardtii* (Green alga) /algae | http://www.phytozome.net-Creinhardtii_169 (DS496108-DS497664) | Merchant et al (2007) |
| [*Chlorella*](http://en.wikipedia.org/wiki/Chlorella) *variabilis* /algae | http://www.ncbi.nlm.nih.gov  (ADIC01000001-ADIC01003810) | Blanc et al (2010) |
| [*Ostreococcus*](http://en.wikipedia.org/wiki/Ostreococcus) *lucimarinus*/algae | http://www.ncbi.nlm.nih.gov  (CP000581-CP000601) | Palenik et al (2007) |
| [*Ostreococcus*](http://en.wikipedia.org/wiki/Ostreococcus) *tauri*/algae | http://www.ncbi.nlm.nih.gov  (NC_014426- NC_014445) | Derelle et al (2006) |
| *Volvox carteri* (Volvox) /algae | http://www.phytozome.net-Vcarteri_199  (GL378323-GL379573) | Prochnik et al (2010) |
| [*Cyanidioschyzon merolae*](http://en.wikipedia.org/wiki/Cyanidioschyzon_merolae) ([Red algae](http://en.wikipedia.org/wiki/Rhodophyta)) /algae | http://www.ncbi.nlm.nih.gov  (AP006483-AP006502) | Matsuzaki et al (2004) |
| *Chondrus crispus* (Irish moss) /algae | http://www.ncbi.nlm.nih.gov  (HG001459-HG002383) | Collén et al (2013) |

**Supplemental Table S2. A Summary of Tandem array (TA) TRIMs in plants**

| Genomes | Number of TA TRIMs | Names of TA TRIMs |
| --- | --- | --- |
| *S. lycopersicum* | 4 | SlyRetroS1, SlyRetroS2, SlyRetroS3, SlyRetroS9 |
| *S.pimpinellifolium* | 4 | SpiRetroS1, SpiRetroS4, SpiRetroS5, SpiRetroS9 |
| *S. tuberosum* | 6 | StuRetroS2, StuRetroS3, StuRetroS4, StuRetroS5, StuRetroS8, StuRetroS9 |
| *C. lanatus* | 1 | ClaRetroS3 |
| *P. mume* | 2 | PmuRetroS2, PmuRetroS4 |
| *Malus x domestica* | 3 | MdoRetroS1, MdoRetroS3, MdoRetroS6 |
| *P. bretschneideri* | 6 | PbrRetroS1, PbrRetro2, PbrRetro3, PbrRetro4, PbrRetro7,PbrRetro8 |
| *F. vesca* | 3 | FveRetroS1, FveRetro2, FveRetro4 |
| *C. sativa* | 3 | CsaRetroS1, Csa-Cassandra, CsaRetroS3 |
| *L. japonicus* | 3 | Lja-Cassandra, LjaRetroS5, LjaRetroS6 |
| *G. max* | 5 | Gma-Cassandra, GmaRetroS2, GmaRetro11, GmaRetro13, GmaRetro40 |
| *C. cajan* | 9 | CcaRetroS1, CcaRetroS2, CcaRetroS4, CcaRetroS5,CcaRetroS6, CcaRetro8,Cca-Cassandra, CcaRetro13, CcaRetro14 |
| *J. curcas* | 5 | Jcu-Cassandra, JcuRetroS2, JcuRetroS3, JcuRetroS6, JcuRetro7 |
| *L. usitatissimum* | 3 | LusRetroS1, LusRetroS4, LusRetroS5 |
| *P. trichocarpa* | 5 | PtrRetroS2, PtrRetroS3, PtrRetro4,PtrRetroS5, PtrRetro6 |
| *A. thaliana* | 2 | At1, Ath-Cassandra |
| *A. lyrata* | 7 | AlyRetroS2, AlyRetroS3, Aly-Cassandra, AlyRetroS11, AlyRetro12, AlyRetroS13, AlyRetroS15 |
| *T. salsuginea* | 7 | TsaRetroS1, TsaRetroS2, TsaRetroS3, TsaRetroS6, TsaRetroS9-Cassandra, TsaRetroS10, TsaRetroS11 |
| *B. rapa* | 6 | Br1, Br4, Bra-Cassandra, BraRetroS5, BraRetroS9, BraRetroS11 |
| *T. parvula* | 1 | Tpa-Cassandra |
| *T. cacao* | 1 | TcaRetroS1 |
| *V. vinifera* | 2 | VviRetroS1, VviRetroS5 |
| *C. sinensis* | 2 | CsiRetroS1, CsiRetroS2 |
| *S. bicolor* | 2 | SbiRetroS8, Sbi-Cassandra |
| *Z. mays* | 4 | Zma-SMART, Zma-Cassandra, ZmaRetroS3, ZmaRetroS5 |
| *S. italica* | 2 | Sit-Cassandra, Sit-SMART |
| *O. sativa*-*indica* | 6 | Osaj-Smart, Osaj-Cassandra, OsajRetroS3, OsajRetroS 10, OsajRetroS 11, OsajRetroS 17 |
| *O. sativa*-*japonica* | 6 | Osai-Smart, Osai-Cassandra, OsaiRetroS3, OsaiRetroS 10, OsaiRetroS 11, OsaiRetroS 17 |
| *O. brachyantha* | 4 | Obr-Smart, ObrRetroS10, ObrRetroS11, Obr-Cassandra |
| *B. distachyon* | 2 | Bdi-SMART, BdiRetroS15 |
| *P. dactylifera* | 3 | PdaRetroS1, PdaRetroS2, PdaRetroS8 |
| *M. acuminata* | 2 | MacRetroS1, MacRetroS2 |
| *S. moellendorffii* | 5 | SmoRetroS1, SmoRetroS2, SmoRetroS4, SmoRetroS5, SmoRetroS7 |
| *V. carteri* | 2 | VcaRetroS3, VcaRetroS4 |
| *C. crispus* | 1 | CcrRetroS1 |

**Supplemental Table S3. Summary of different types of TA-TRIMs in *Z. mays* genome**

| TA-TRIM | Zma-SMART | Zma-Cassandra | ZmaRetroS3 | ZmaRetroS5 | All |
| --- | --- | --- | --- | --- | --- |
| L_3_I_2_ | 16 | 34 | 10 | 3 | 63 |
| L_4_I_2_ | 2 | 4 |  |  | 6 |
| R- L_4_I_2_ | 5 | 1 |  |  | 6 |
| L_4_I_3_ |  | 2 | 3 | 2 | 7 |
| L_5_I_4_ | 2 | 1 |  | 1 | 4 |
| other |  | 4 | 3 |  | 7 |
| Total | 25 | 46 | 16 | 6 | 93 |

**Supplemental Table S4. Summary of TRIM-related genes in 14 plant genomes**

| Genome | Exon | | Intron | | 1.5kb upstream | | All | |
| --- | --- | --- | --- | --- | --- | --- | --- | --- |
|  | Number | Fraction (%) | Number | Fraction (%) | Number | Fraction (%) | Number | Fraction (%) |
| *S. lycopersicum* | 65 | 0.7 | 2,296 | 25.1 | 1,033 | 11.3 | 3,394 | 37.0 |
| *S. tuberosum* | 723 | 5.8 | 1,725 | 13.8 | 1,141 | 9.1 | 3,589 | 28.8 |
| *G. max* | 276 | 2.7 | 3,444 | 34.1 | 719 | 7.1 | 4,439 | 43.9 |
| *C. cajan* | 354 | 1.7 | 3,370 | 16.1 | 1,840 | 8.8 | 5,564 | 26.6 |
| *M. truncatula* | 938 | 11.1 | 1,151 | 13.7 | 1,662 | 19.7 | 3,751 | 44.6 |
| *P. trichocarpa* | 67 | 1.3 | 447 | 8.4 | 936 | 17.7 | 1,450 | 27.4 |
| *A. thaliana* | 27 | 3.1 | 28 | 3.2 | 110 | 12.6 | 165 | 18.8 |
| *A. lyrata* | 30 | 1.7 | 217 | 12.6 | 424 | 24.6 | 671 | 38.9 |
| *V. vinifera* | 94 | 1.1 | 3,553 | 40.0 | 749 | 8.4 | 4,396 | 49.4 |
| *Z. mays* | 87 | 1.0 | 1419 | 15.7 | 361 | 4.0 | 1,867 | 20.7 |
| *S. bicolor* | 9 | 0.3 | 521 | 17.8 | 156 | 5.3 | 686 | 23.5 |
| *B. distachyon* | 18 | 1.2 | 384 | 25.5 | 204 | 13.5 | 606 | 36.0 |
| *M. acuminata* | 106 | 2.3 | 869 | 19.0 | 542 | 11.8 | 1,517 | 33.1 |
| *V. carteri* | 84 | 4.1 | 559 | 27.2 | 266 | 13.0 | 909 | 44.3 |
| **Average** | 206 | 2.7 | 1427 | 19.4 | 725 | 11.9 | 2357 | 34.1 |

Note: fraction means the percentage of the gene-related TRIMs to the total TRIMs.

**Supplemental Table S5**. **Summary of TRIMs and other TEs located in and near annotated genes in two genomes**

| Genome | | Exon | | Intron | | 1.5 Kb Upstream | | Total | |
| --- | --- | --- | --- | --- | --- | --- | --- | --- | --- |
|  |  | Number | Percentage (%) | Number | Percentage (%) | Number | Percentage (%) | Number | Percentage (%) |
| *Z. mays* | TRIM | 87 | 1.0 | 1419 | 15.7 | 361 | 4.0 | 1,867 | 20.7 |
|  | Ty3-gypsy | 1,856 | 0.25 | 13,526 | 1.83 | 14,823 | 2.01 | 30,205 | 4.09 |
|  | Ty1-copia | 1,210 | 0.25 | 17,760 | 3.60 | 11,949 | 2.42 | 30,919 | 6.27 |
|  | MITEs | 3,772 | 3.94 | 13,579 | 14.20 | 18,147 | 18.98 | 35,498 | 37.12 |
| *G. max* | TRIM | 276 | 2.7 | 3,444 | 34.1 | 719 | 7.1 | 4,439 | 43.9 |
|  | Ty3-gypsy | 5,163 | 1.58 | 15,923 | 4.89 | 16,957 | 5.21 | 38,043 | 11.68 |
|  | Ty1-copia | 3,293 | 1.86 | 14,818 | 8.35 | 11,087 | 6.25 | 29,198 | 16.46 |
|  | MITEs | 600 | 1.44 | 8,071 | 19.43 | 7,004 | 16.87 | 15,675 | 37.74 |

**Supplemental Table S6. Comparison of TRIM-related genes and non-TRIM-related**

**genes in *G. max* and *Z. mays***

| Genome | Genes (Number) | Exon count | Exon size  (bp) | Intron size  (bp) | Gene size  (bp) |
| --- | --- | --- | --- | --- | --- |
| *G. max* | TRIM-related (2494) | 12.2 | 2278.3 | 6645.0 | 8923.3 |
|  | Non-TRIM-related (43873) | 5.9 | 1523.3 | 2053.7 | 3579.0 |
| *Z. mays* | TRIM-related (961) | 10.1 | 2132.8 | 12444.6 | 14577.4 |
|  | Non-TRIM-related (38695) | 5.1 | 1529.4 | 2546.9 | 4076.3 |

**Supplemental Table S7. Comparison of Comparison of TRIM density in different sizes of genes in *G. max* and *Z. mays***

| Genome | Gene (Number) | Insertion events/per gene | Insertion events/per Kb |
| --- | --- | --- | --- |
| *G. max* | Small gene (9273) | 21/9273=0.0023 | 21/7621.279=0.0028 |
|  | Large gene (9273) | 1554/9273=0.1676 (72.9:1) | 1554/84971.600=0.0183 (6.54:1) |
| *Z. mays* | Small gene (7931) | 19/7931=0.0024 | 19/4560.927=0.0041 |
|  | Large gene (7931) | 1005/7931=0.1267(52.8:1) | 1005/119522.749=0.0084 (2.0:1) |

Note: The TRIM density = TRIM numbers/gene numbers (or the coverage of the genes) for small and larger genes. The TRIM density between larger and smaller genes were significantly different indicated by Pearson's Chi-squared test (p value < 0.05).

**Supplemental Table S8. Comparison of homologous genes between *G. max* and *Z. mays* and two related genomes**

| Genome | Genes | Query genes | | | Homologs 1 | | | Homologs 2 | | |
| --- | --- | --- | --- | --- | --- | --- | --- | --- | --- | --- |
|  |  | Exon count | Exon size  (bp) | Intron size  (bp) | Exon count | Exon size  (bp) | Intron size  (bp) | Exon count | Exon size  (bp) | Intron size  (bp) |
| *G. max* | TRIM-related (2383) | 12.5 | 2321.7 | 6823.1 | 12.1 | 2000.0 | 7054.1 | 12.5 | 2583.2 | 6848.2 |
|  | Non-TRIM-related (22863) | 6.4 | 1609.2 | 2236.2 | 6.3 | 1320.6 | 2696.0 | 6.5 | 1837.3 | 2482.3 |
| *Z. mays* | TRIM-related (817) | 10.9 | 2243.7 | 13956.5 | 11.4 | 2175.3 | 6546.8 | 10.3 | 2696.8 | 4290.6 |
|  | Non-TRIM-related (22258) | 6.3 | 1772.4 | 3664.3 | 6.9 | 1738.9 | 2745.5 | 6.8 | 2085.4 | 2294.6 |

Note: Homolog 1 and 2 for *G. max* represent the genes in *C. cajan* and *P. vulgaris* while the Homolog 1 and 2 for *Z. mays* mean the genes in *S. bicolor* and *O. sativa*.

**Supplemental Table S9. Summary of TRIMs and other TEs located in and near syntenic genes in two genomes**

| Genome | | Exon | | Intron | | 1.5 Kb Upstream | | Total | |
| --- | --- | --- | --- | --- | --- | --- | --- | --- | --- |
|  |  | Number | Percentage (%) | Number | Percentage (%) | Number | Percentage (%) | Number | Percentage (%) |
| *Z. mays* | TRIM | 30 | 0.33 | 678 | 7.50 | 125 | 1.38 | 833 | 9.22 |
|  | Ty3-gypsy | 354 | 0.05 | 4696 | 0.64 | 3851 | 0.52 | 8901 | 1.20 |
|  | Ty1-copia | 336 | 0.07 | 7033 | 1.43 | 4221 | 0.86 | 11590 | 2.35 |
|  | MITEs | 1907 | 1.99 | 7022 | 7.34 | 7892 | 8.25 | 16821 | 17.59 |
| *G. max* | TRIM | 139 | 1.38 | 2493 | 24.68 | 429 | 4.25 | 3061 | 30.30 |
|  | Ty3-gypsy | 1480 | 0.45 | 10559 | 3.24 | 10169 | 3.12 | 22208 | 6.82 |
|  | Ty1-copia | 1087 | 0.61 | 10079 | 5.68 | 6469 | 3.65 | 17635 | 0.99 |
|  | MITEs | 274 | 0.66 | 5680 | 13.68 | 4909 | 11.82 | 10863 | 26.16 |

**Supplemental Table S10.**  **Comparison of the syntenic genes related to different transposon groups in two genomes**

| Genome | Transposons | Genes (Number) | Exon count | Exon size  (bp) | Intron size  (bp) | Gene size  (bp) |
| --- | --- | --- | --- | --- | --- | --- |
| *G. max* | TRIM | Related (1770) | 12.3 | 2280.4 | 6725.0 | 9005.4 |
|  |  | No-related (29083) | 5.8 | 1493.7 | 2006.0 | 3499.8 |
|  | Ty3-gypsy | Related (6727) | 9.1 | 1864.0 | 4433.9 | 6298.9 |
|  |  | No-related (24126) | 5.3 | 1448.2 | 1675.3 | 3123.5 |
|  | Ty1-copia | Related (5902) | 9.6 | 1933.0 | 4903.6 | 6836.5 |
|  |  | No-related (24951) | 5.3 | 1445.6 | 1655.4 | 3101.0 |
|  | MITEs | Related (3619) | 8.8 | 1809.3 | 4118.1 | 5927.4 |
|  |  | No-related (27234) | 5.8 | 1502.9 | 2032.1 | 3535.0 |
| *Z. mays* | TRIM | Related (771) | 8.9 | 1912.6 | 9945.8 | 11858.4 |
|  |  | No-related (22899) | 4.5 | 1561.0 | 2039.8 | 3600.7 |
|  | Ty3-gypsy | Related (2165) | 7.0 | 1719.6 | 9264.5 | 10984.0 |
|  |  | No-related (21505) | 4.4 | 1557.6 | 1595.9 | 3153.5 |
|  | Ty1-copia | Related (2260) | 7.9 | 1846.3 | 9835.7 | 11682.0 |
|  |  | No-related (21410) | 4.3 | 1543.5 | 1501.6 | 3045.1 |
|  | MITEs | Related (7427) | 6.3 | 1700.3 | 4317.7 | 6018.0 |
|  |  | No-related (16243) | 3.9 | 1513.9 | 1373.5 | 2887.4 |

**Supplemental Table S11. Comparison of sequence evolutionary rates between TRIM-related genes and non-TRIM related genes**

|  | *Glycine max*^a^ | | |  | *Zea mays*^b^ | | |
| --- | --- | --- | --- | --- | --- | --- | --- |
|  | TRIM related | non-TRIM related | *P-value*^c^ |  | TRIM related | non-TRIM related | *P-value*^c^ |
| *Ka* | 0.0389 | 0.0395 | 0.2461 |  | 0.0298 | 0.0338 | 5.4 x 10^-07^ |
| *Ks* | 0.2154 | 0.2447 | < 2.2 x 10^-16^ |  | 0.1366 | 0.2633 | < 2.2 x 10^-16^ |
| *Ka/Ks* | 0.1918 | 0.2313 | 5.3 x 10^-07^ |  | 0.2492 | 0.9572 | < 2.2 x 10^-16^ |

Note: ^a^ *Ka* and *Ks* were calculated by pairwise comparison between *G. max* and *P. vulgaris.* ^b^ *Ka* and *Ks* were calculated by pairwise comparison between *Z. mays* and *S. bicolor.* ^c^ Wilcoxon rank sum test between TRIM related and non-TRIM related genes.

**Supplemental Table S12. List of 7 plant TRIMs containing gene sequences identified in this study**

| Plant genome | TRIM name | GenBank accession No. of TRIM | TRIM position | Complete copy of TRIMs | TRIM  size (bp) | Function of host genes | Related EST/cDNA |
| --- | --- | --- | --- | --- | --- | --- | --- |
| *M.truncatula* | MtrRetroS2 | NC_016408 | 16817079- 16818427 | 4 | 1348 | hypothetical protein | [EX528321](http://www.ncbi.nlm.nih.gov/nucleotide/157980049?report=genbank&log$=nuclalign&blast_rank=1&RID=HRX397VH013) |
| *G. max* | GmaRetroS1  GmaRetroS4  GmaRetroS10  GmaRetroS11  GmaRetroS15  GmaRetroS28 | [NW_003722731](http://www.ncbi.nlm.nih.gov/nucleotide/353336030?report=genbank&log$=nuclalign&blast_rank=1&RID=JA6CU3EW013)  NW_003722741  NW_003722750  NW_003722733  NW_003722733  NW_003722742 | 5520453- 5521882  26859984- 26861427  36828364- 36829790  4976728- 4977993  4996492- 4997940  7326874- 7328045 | 1  5  5  12  2  1 | 1430  1444  1427  1266  1449  1172 | [cysteinyl-tRNA synthetase-like](http://blast.ncbi.nlm.nih.gov/Blast.cgi#alnHdr_356536300)  uncharacterized protein  LRR receptor-like serine/threonine-protein kinase  uncharacterized protein  [LRR receptor-like serine/threonine-protein kinase RPK2-like](http://blast.ncbi.nlm.nih.gov/Blast.cgi#alnHdr_356527945)  uncharacterized protein  uncharacterized protein  casein kinase I isoform delta-like  receptor-like serine/threonine-protein kinase SD1-8 | [XM_003555862](http://www.ncbi.nlm.nih.gov/nucleotide/356575565?report=genbank&log$=nuclalign&blast_rank=1&RID=HTWCAEWY01R)  XM_006584122  XM_006599954  [XM_003527608](http://www.ncbi.nlm.nih.gov/nucleotide/356517967?report=genbank&log$=nuclalign&blast_rank=1&RID=HU20JPXB013)  XM_006596579  XM_003532044  XM_003522125  XM_003516429  XM_003532518 |

**Supplemental Table S13.** **The numbers of TRIMs that were inserted into genic regions in *G. max* and *Z. mays***

| Genome | Type | TRIM family | 21nt siRNA^a^ | 24nt siRNA^a^ | 21nt & 24nt siRNA^b^ |
| --- | --- | --- | --- | --- | --- |
| *G. max* | Type I | Gma-Cassandra | 378 (25.8%) | 930 (63.5%) | 360 (24.6%) |
|  |  | GmaRetroS2 | 479 (24.4%) | 1182 (60.2%) | 461 (23.5%) |
|  |  | GmaRetroS11 | 36 (20.3%) | 81 (45.8%) | 31 (17.5%) |
|  |  | GmaRetroS12 | 157 (36.9%) | 290 (68.1%) | 152 (35.7%) |
|  |  | GmaRetroS13 | 179 (20.4%) | 396 (45.2%) | 164 (18.7%) |
|  |  | GmaRetroS40 | 307 (11.4%) | 839 (31.1%) | 266 (9.8%) |
|  | Type II | GmaRetroS1 | 44 (7.0%) | 199 (31.6%) | 34 (5.4%) |
|  |  | GmaRetroS3 | 13 (5.1%) | 71 (28.1%) | 12 (4.7%) |
|  |  | GmaRetroS4 | 15 (6.5%) | 44 (19.0%) | 12 (5.2%) |
|  |  | GmaRetroS10 | 18 (6.8%) | 83 (32.8%) | 14 (5.3%) |
|  |  | GmaRetroS15 | 39 (6.0%) | 191 (29.5%) | 27 (4.2%) |
|  |  | GmaRetroS27 | 16 (7.2%) | 67 (30.0%) | 14 (6.3%) |
|  |  | GmaRetroS28 | 16 (5.5%) | 94 (32.5%) | 12 (4.2%) |
|  | Type III | GmaRetroS14 | 8 (1.7%) | 81 (16.8%) | 2 (0.4%) |
|  |  | GmaRetroS25 | 6 (0.8%) | 25 (3.2%) | 3 (0.4%) |
|  |  | GmaRetroS38 | 0 (0%) | 14 (2.3%) | 0 (0%) |
|  |  | GmaRetroS39 | 11 (1.6%) | 87 (12.9%) | 7 (1.0%) |
| *Z. mays* | Type I | Zma-SMART | 115 (8.9%) | 555 (42.9%) | 96 (7.4%) |
|  |  | ZmaRetroS3 | 52 (15.4%) | 155 (46.0%) | 49 (14.5%) |
|  |  | ZmaRetroS5 | 44 (29.7%) | 92 (62.2%) | 44 (29.7%) |
|  | Type II | Zma-Cassandra | 220 (3.8%) | 973 (16.8%) | 204 (3.5%) |
|  |  | ZmaRetroS4 | 13 (3.1%) | 94 (22.1%) | 10 (2.4%) |
|  |  | ZmaRetroS11 | 12 (2.7%) | 89 (19.7%) | 9 (2.0%) |

^a^ Number of TRIMs that were targeted by 21nt or 24nt siRNA, the percentage indicates the portion among total TRIM across the genome.

^b^ Number of TRIMs that were targeted by both 21nt and 24nt siRNA, the percentage indicates the portion among total TRIM across the genome.

**Supplemental Table S14. The portions of methylated genes in TRIM-related genes (TRGs) and non-TRIM-related genes (NTRGs)**

|  | *G. max* |  |  | *Z. mays* |  |
| --- | --- | --- | --- | --- | --- |
|  | TRG | NTRG |  | TRG | NTRG |
| Unmethylated^a^ | 532 (21.4%) | 28,999 (66.4%) |  | 145 (15.1%) | 20,432 (54.4%) |
| CG body-methylated^b^ | 1,206 (48.5%) | 8,656 (19.8%) |  | 187 (19.5%) | 3,433 (9.1%) |
| C methylated^c^ | 681 (27.4%) | 4,786 (11.0%) |  | 617 (64.3%) | 13,217 (35.2%) |
| Total | 2,489 | 43,654 |  | 959 | 37,586 |

^a^ *P_CG_* > 0.95 and not (*P_CHG_* < 0.05 or *P_CHH_* < 0.05)

^b^ *P_CG_* < 0.05 and not (*P_CHG_* < 0.05 or *P_CHH_* < 0.05)

^c^ *P_CHG_* < 0.05 or *P_CHH_* < 0.05

**Supplemental Table S15. The numbers of TRIMs that were inserted into genic regions in *G. max* and *Z. mays***

| Genome | Type | TRIM family | CG body-methylated^a^ | C methylated^b^ | Total^c^ |
| --- | --- | --- | --- | --- | --- |
|  | Type I | Gma-Cassandra | 18 (22.0%) | 60 (73.2%) | 82 (5.6%) |
|  |  | GmaRetroS2 | 42 (15.4%) | 140 (51.3%) | 273 (13.9%) |
|  |  | GmaRetroS11 | 0 (0%) | 17 (68.0%) | 25 (14.1%) |
|  |  | GmaRetroS12 | 11 (16.7%) | 43 (65.2%) | 66 (15.5%) |
|  |  | GmaRetroS13 | 136 (39.3%) | 113 (32.7%) | 346 (39.5%) |
|  |  | GmaRetroS40 | 761 (53.9%) | 380 (26.9%) | 1,412 (52.3%) |
|  | Type II | GmaRetroS1 | 16 (20.3%) | 55 (69.6%) | 79 (12.5%) |
|  |  | GmaRetroS3 | 5 (19.2%) | 15 (57.7%) | 26 (10.3%) |
|  |  | GmaRetroS4 | 5 (20.0%) | 12 (48.0%) | 25 (10.8%) |
|  |  | GmaRetroS10 | 2 (7.1%) | 21 (75.0%) | 28 (10.6%) |
|  |  | GmaRetroS15 | 5 (6.1%) | 64 (78.0%) | 82 (12.7%) |
|  |  | GmaRetroS27 | 3 (16.7%) | 10 (55.6%) | 18 (8.1%) |
|  |  | GmaRetroS28 | 2 (6.3%) | 22 (68.8%) | 32 (11.1%) |
|  | Type III | GmaRetroS14 | 123 (49.8%) | 108 (43.7%) | 247 (51.2%) |
|  |  | GmaRetroS25 | 310 (61.0%) | 87 (17.1%) | 508 (64.5%) |
|  |  | GmaRetroS38 | 284 (64.4%) | 68 (15.4%) | 441 (71.6%) |
|  |  | GmaRetroS39 | 198 (48.3%) | 124 (30.2%) | 410 (60.7%) |
|  | Type I | Zma-SMART | 166 (22.4%) | 453 (61.1%) | 742 (57.3%) |
|  |  | ZmaRetroS3 | 11 (19.3%) | 41 (71.9%) | 57 (16.9%) |
|  |  | ZmaRetroS5 | 2 (12.5%) | 13 (81.3%) | 16 (10.8%) |
|  | Type II | Zma-Cassandra | 21 (9.9%) | 172 (80.8%) | 213 (3.7%) |
|  |  | ZmaRetroS4 | 0 (0%) | 15 (88.2%) | 17 (4.0%) |
|  |  | ZmaRetroS11 | 39 (15.3%) | 201 (78.8%) | 255 (56.4%) |

^a^ TRIMs that were inserted into CG body-methylated genes, the percentage indicates the portion among total genic insertion.

^b^ TRIMs that were inserted into C methylated genes, the percentage indicates the portion among total genic insertion.

^c^ TRIMs that were inserted into genes, the percentage indicates the portion among total insertion.

**Supplemental Table S16. Plant TRIMs and their putative autonomous LTR retrotransposons**

| Genome | TRIMs | | | | Putative autonomous elements | | | | | |
| --- | --- | --- | --- | --- | --- | --- | --- | --- | --- | --- |
|  | Name | Position | Element size (bp) | LTR  size (bp) | Name | Position | Super-familiy | Element size (bp) | LTR size (bp)  (Identical to TRIM, %) | Retrotransposase size (aa) |
| *S. lycopersicum* | SlyRetroS4 | [AC247212](http://www.ncbi.nlm.nih.gov/nucleotide/418204002?report=genbank&log$=nuclalign&blast_rank=1&RID=JMHKMZ2G014)  (145904-146233) | 330 | 115 | SlyLTRA4 | EF647605  (72251-77298 ) | Ty1-copia | 5048 | 116 (95) | 1115 |
| *P. trichocarpa* | PtrRetroS2 | [AARH01000047](http://www.ncbi.nlm.nih.gov/nucleotide/114383907?report=genbank&log$=nuclalign&blast_rank=1&RID=JMKGFTSN016) (2969-2240) | 731 | 190 | PtrLTRA2 | NW_001492710  (256430- 262074 | Ty1-copia | 5645 | 190 (97) | 1013 |
| *V. vinifera* | VviRetroS5 | [CAAP03011320 (28901-30016)](http://www.ncbi.nlm.nih.gov/nucleotide/270214723?report=genbank&log$=nuclalign&blast_rank=1&RID=JMM8MAMP016) | 1116 | 223 | VviLTRA5 | NW_002238140  (571224-567858) | Ty1-copia | 3367 | 224 (95) | 384 |
| *P. bretschneideri* | PbrRetroS6 | [AJSU01017719](http://www.ncbi.nlm.nih.gov/nucleotide/422964744?report=genbank&log$=nuclalign&blast_rank=1&RID=3H4U750E01R)  (13862-15248) | 1387 | 258 | PbrLTRA6 | [AJSU01000328](http://www.ncbi.nlm.nih.gov/nucleotide/422986874?report=genbank&log$=nuclalign&blast_rank=1&RID=3H5SHGWZ01R)  (7241-14551) | Ty1-copia | 7316 | 257 (93) | 466 |
| *C. arietinum* | CarRetroS1 | [ANPC01000069](http://www.ncbi.nlm.nih.gov/nucleotide/434889991?report=genbank&log$=nuclalign&blast_rank=1&RID=3H92MJJJ01R)  (164228-165696) | 1469 | 278 | CarLTRA1 | CM001770  (19032686-19036138) | Ty1-copia | 3453 | 284 (88) | 549 |
| *C. arietinum* | CarRetroS2 | ANPC01004602 (21622- 22878) | 1257 | 185 | CarLTRA2 | CM001767  (47372860-47378133) | Ty1-copia | 5274 | 185(79) | 363 |
| *G. max* | GmaRetroS2 | ACUP01004189 (130251-130764) | 514 | 181 | GmaLTRA2 | ACUP01004178 (141016-136827) | Ty1-copia | 4190 | 181(93) | 793 |
| *S. italica* | SitLTRS5 | [AGNK01003787](http://www.ncbi.nlm.nih.gov/nucleotide/387425342?report=genbank&log$=nuclalign&blast_rank=1&RID=JMMZC51F016) (84609 -85532) | 924 | 133 | SitLTRA5 | [AGNK01001549](http://www.ncbi.nlm.nih.gov/nucleotide/387430373?report=genbank&log$=nuclalign&blast_rank=7&RID=HV2RHT7601R)  (5190- 10584) | Ty1-copia | 5395 | 131 (89) | 1409 |
| *O. sativa*  Japonica | OsajRetroS10 | [AP002861](http://www.ncbi.nlm.nih.gov/nucleotide/10800055?report=genbank&log$=nuclalign&blast_rank=1&RID=JN9CY69J01R)  (29390-29797) | 408 | 115 | OsajLTRA10 | [AP003234](http://www.ncbi.nlm.nih.gov/nucleotide/21901973?report=genbank&log$=nuclalign&blast_rank=1&RID=HWPSV1S2013)  (140844- 149347) | Ty1-copia | 8504 | 115 (97) | 1577 |
| *O. sativa* -Indica | OsaiRetroS10 | [AAAA02000568](http://www.ncbi.nlm.nih.gov/nucleotide/54312882?report=genbank&log$=nuclalign&blast_rank=4&RID=JNJN7PAK01R)  (26189-26596) | 408 | 115 | OsaiLTRA10 | CM000126  (29314550- 29322610) | Ty1-copia | 8061 | 115 (97) | 1431 |
| *S. moellendorffii* | SmoRetroS4 | [ADFJ01000100](http://www.ncbi.nlm.nih.gov/nucleotide/299479955?report=genbank&log$=nuclalign&blast_rank=1&RID=JNFV6P6C01R) (60741-60349) | 393 | 104 | SmoLTRA4 | [ADFJ01000078](http://www.ncbi.nlm.nih.gov/nucleotide/299479977?report=genbank&log$=nuclalign&blast_rank=1&RID=JNGEC0G101R)  (22401-26773) | Ty3-gypsy | 4373 | 104 (98) | 1218 |

**Supplemental Table S17. List of primers used for PCR and RT-PCR analysis**

| Primer name | Forward primer (5-3’) | Reverse primer (5-3’) | Note |
| --- | --- | --- | --- |
| OsaA10RT | AGCATGGTGATAATCAACTGTT | GTATACTTCGTTTGATGCACA | RT- PCR of OsajLTRA10 |
| Actin | CAAGGCCAATCGTGAGAA | AGCAATGCCAGGGAACATAGT | RT- PCR of OsajLTRA10 |
| P1 | GGCAACCTAATGGTGGTTACA | GGACAGATTTCTGTGGGTCAA | New insertions in rice |
| P2 | AAGAGGAAGGTGAAGGACGAG | TACCGGCAAACAATTGAACTC | New insertions in rice |
| P3 | TGCCAATCTAAAACCAGGATG | AGACAGAGGGAGAAGGAGCTG | New insertions in rice |
| Zm1 | AGGTTCCCATTTCTTGTTGAA | GATGATGATGATGATGCCACA | TA-TRIM in maize |
| Zm3 | CCCAAACATTGCTAGCTTGA | CACCCCGTTTGTTGCTTTAT | TA-TRIM in maize |
| Zm6 | GGTTCGGGGGAAAAATAAAA | GTCACTAGGTCGCTGGTTCG | TA-TRIM in maize |
| Zm7 | GCAAGGGTGTGCCTATGTAGA | TTCTTTGGTTATTTTGTCCTTGC | TA-TRIM in maize |


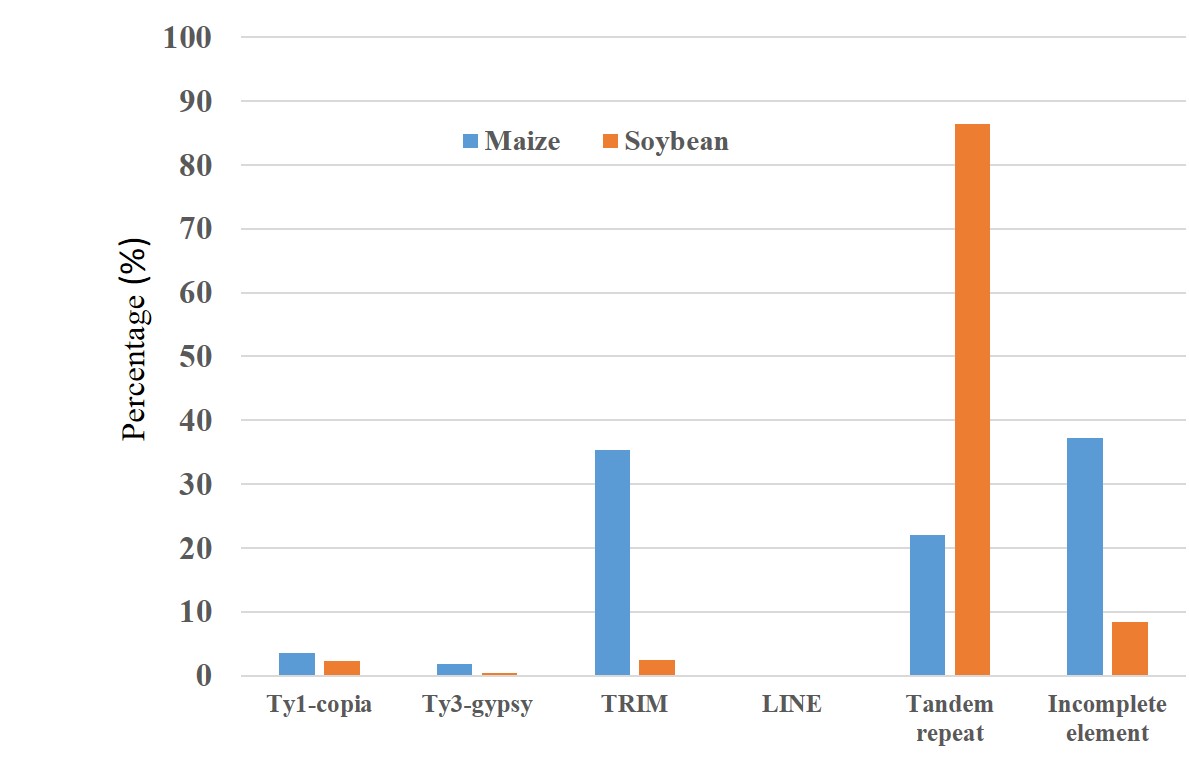


**Supplemental Figure S1. A classification of predicted sequences with LTR_FINDER in maize and soybean.** The predicted sequences are classified into six groups, TRIMs, Ty1 and Ty3 LTR retrotransposons, non LTR retroelement, tandem repeat and incomplete elements including the sequences with gaps.


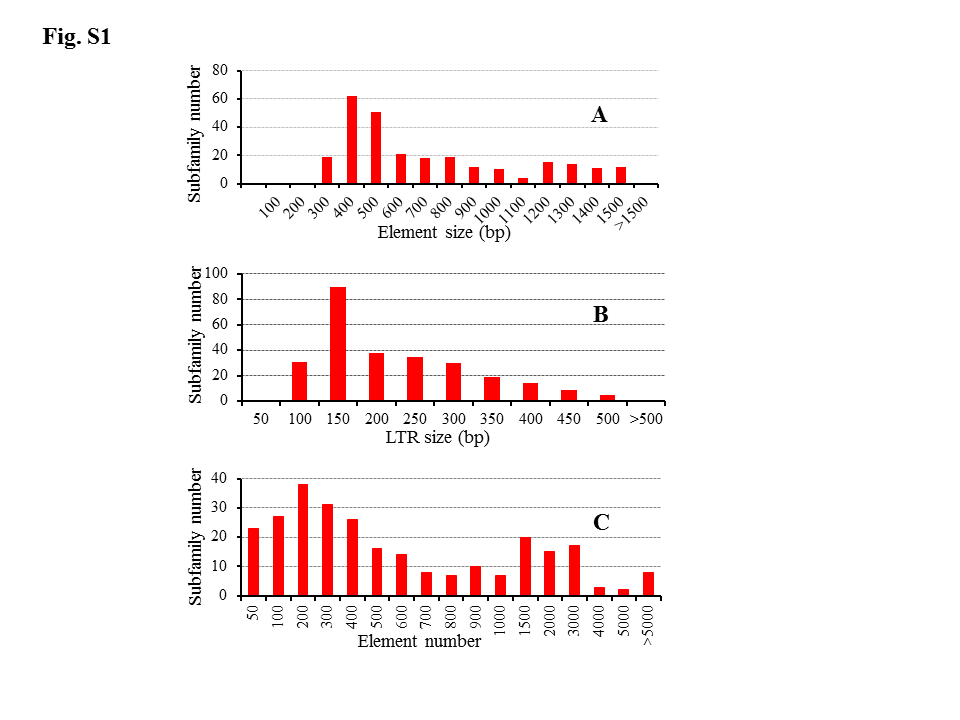


**Supplemental Figure S2. A summary of element sizes (A), LTR sizes (B) and copy numbers (C) of 289 TRIM subfamilies.**

**
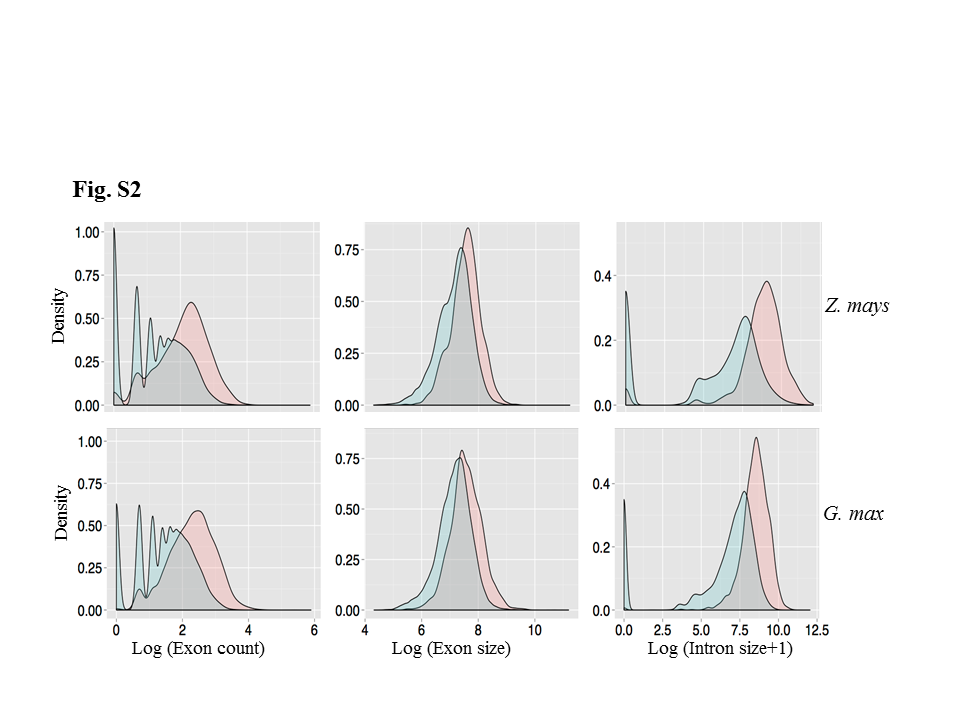
Supplemental Figure S3. Variation in exon count, exon and intron sizes in *G. max* and *Z. mays*.** Red and blue represent TRIM-related and non-TRIM-related genes, respectively.


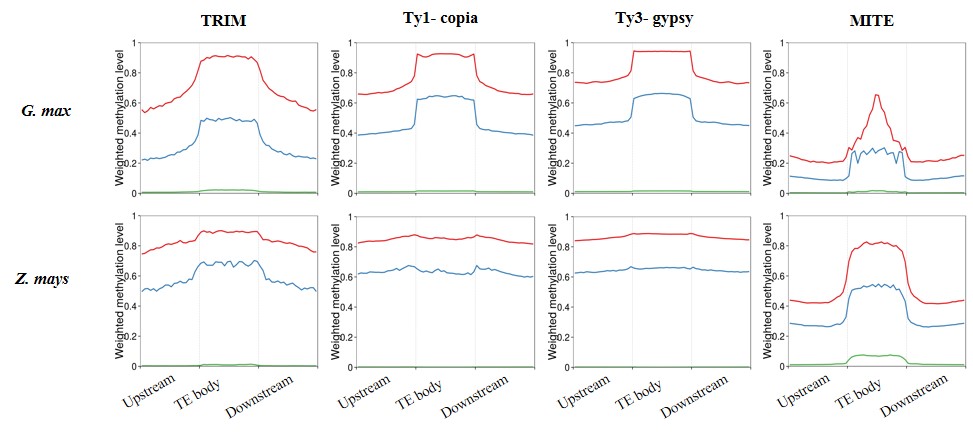


**Supplemental Figure S4. Methylation patterns of TRIMs and other TEs in *G. max* and *Z. mays*.**


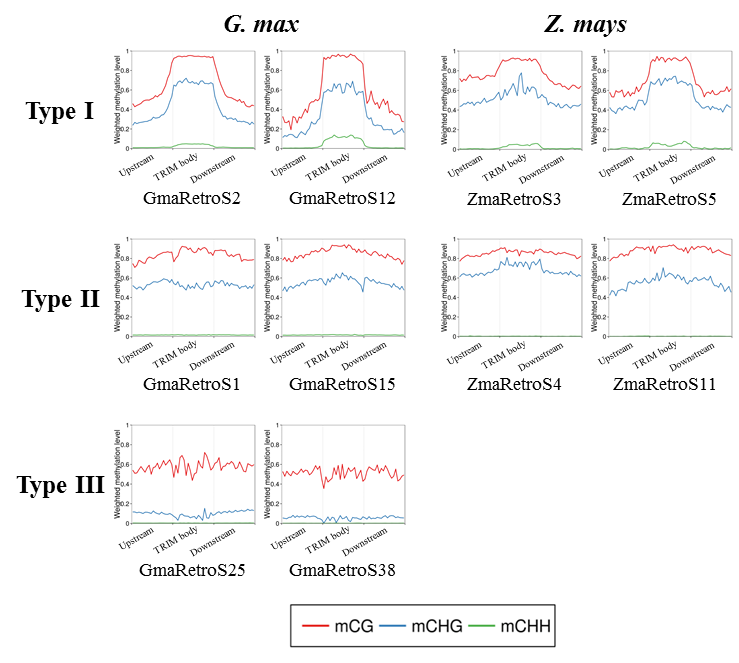


**Supplemental Figure S5. Methylation patterns of three TRIM types (Type I, II and III) in *G. max* and *Z. mays*. Type III was not found in *Z. mays*.**

**
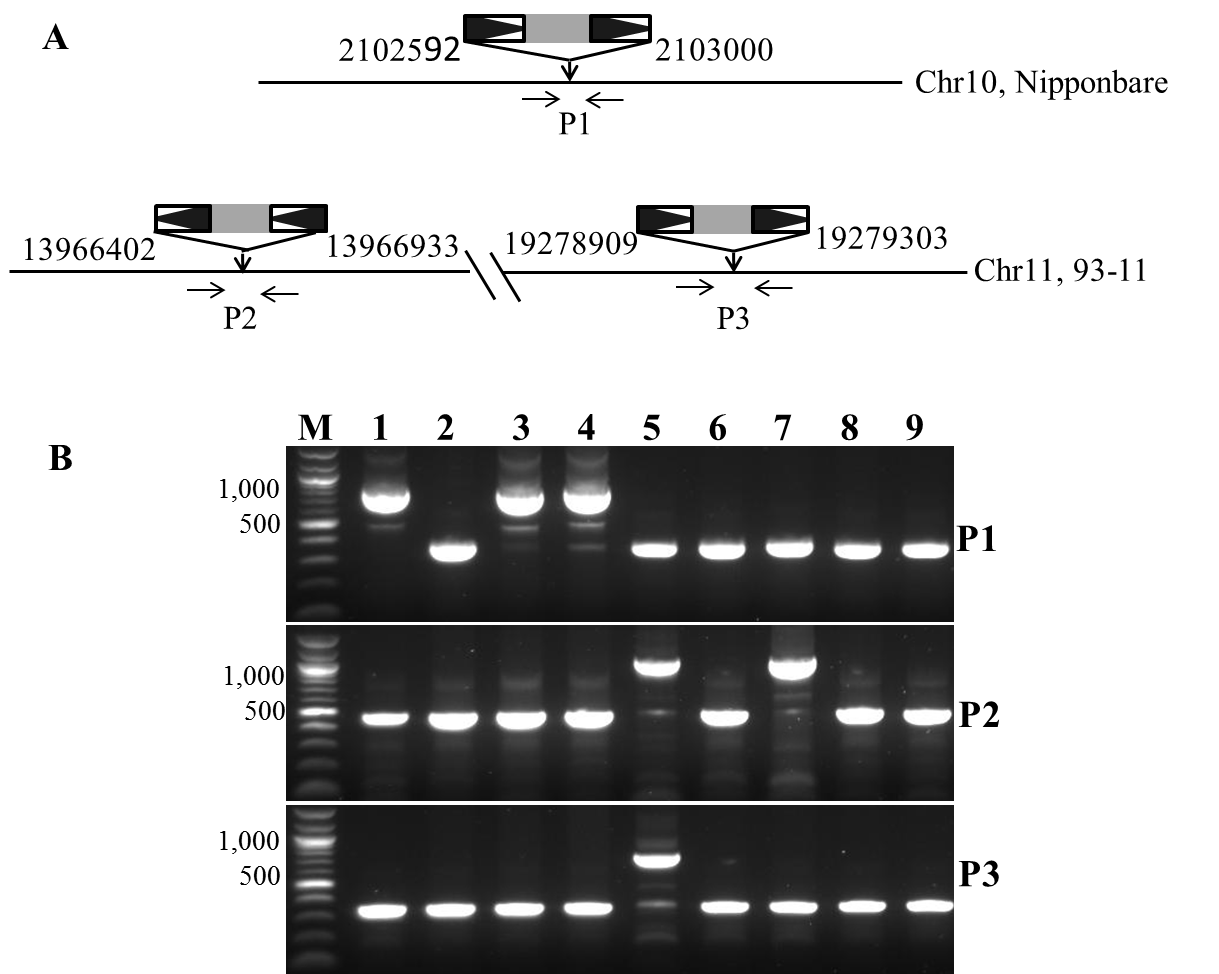
**

**Supplemental Figure S6. New insertions of a TRIM family in rice.** A. One and two new insertions in chromosome 10 of Nipponbare and chromosome 11 of 93-11. Arrows indicate the PCR primers used to amplify TRIMs and flanking sequences. **B.** PCR validation of three new insertions. Lanes1-4 represent four *japonica* rice cultivars, Nipponbare, Kitaaki, Azucena and Moroberkan, 5-7 indicate three *indica* rice cultivars, 93-11, IR36 and IR64 and 9-10 are two wild rice species, *O. nivara* and *O. rufipogon* .

**
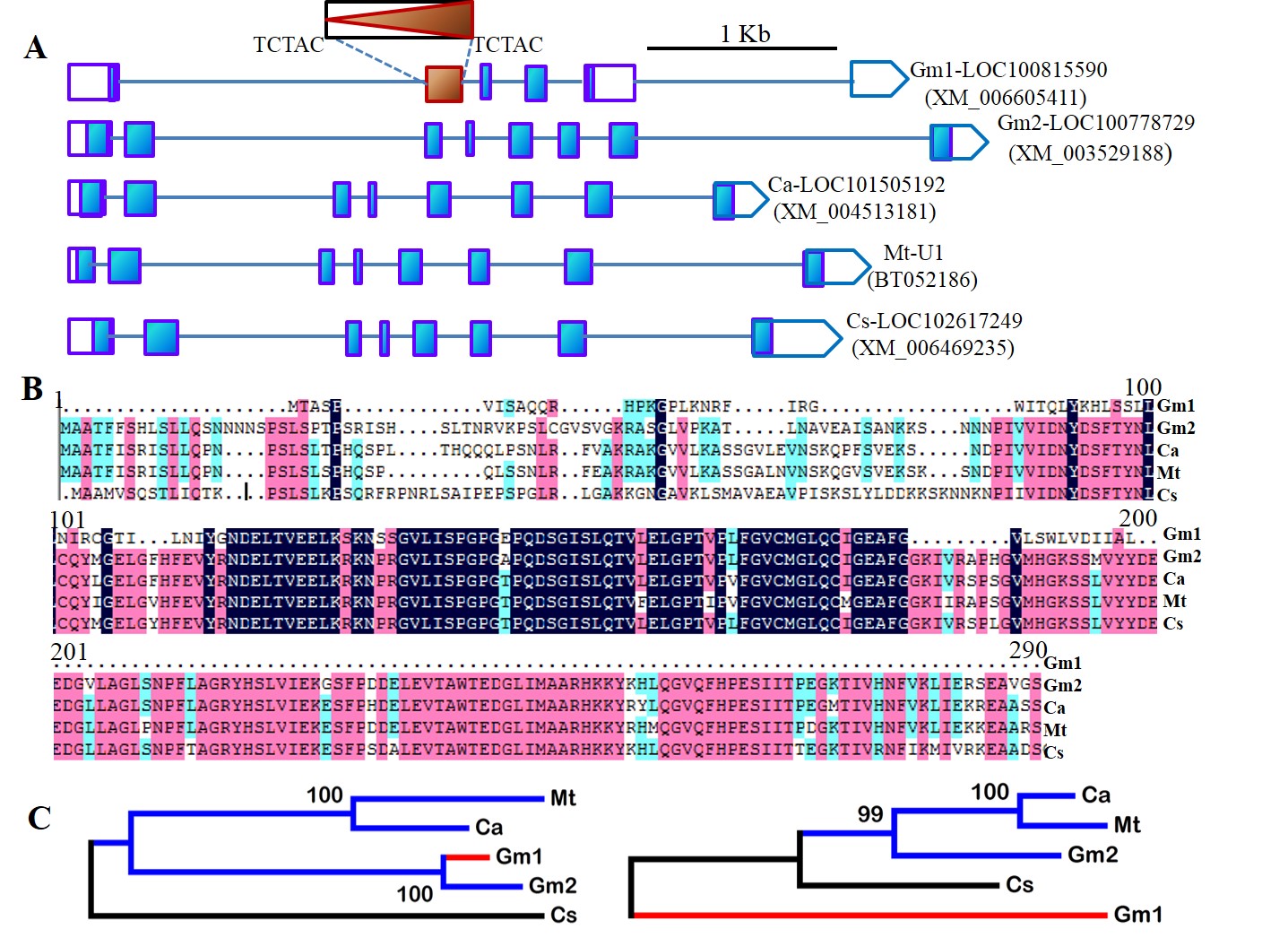
Supplemental Figure S7. Comparison of gene structures and sequences from a TRIM-related gene in *G. max* and homologous genes.** A solo-LTR of a TRIM GmaRetroS12 in *G. max* serves as an exon of LOC100815590 (Gm1). Comparison to the paralogous gene LOC100778729 (Gm2) and homologous genes, LOC101505192 (Ca) from *C. arietinum*, an unknown gene we called U1 (Mt) from *M. truncatula* and LOC102617249 (Cs) from *C. sinensis*, LOC100815590 has a unique exon marked in brown and a differing gene structure. **B.** Alignment of proteins (aligned by DNAman, wwwlynnon.com) encoded by LOC100815590 and homologs, LOC100815590 encodes 121-aa protein whereas others encode 270-283 aa proteins. C. Phylogenetic tree built with gene DNA sequences (left) and proteins (right). The TRIM-related gene in *G. max* is red and the homologous gene in *C. sinensis* is black, used as outgroup sequence. All the gene models were supported by cDNAs (accession numbers in parentheses).

**
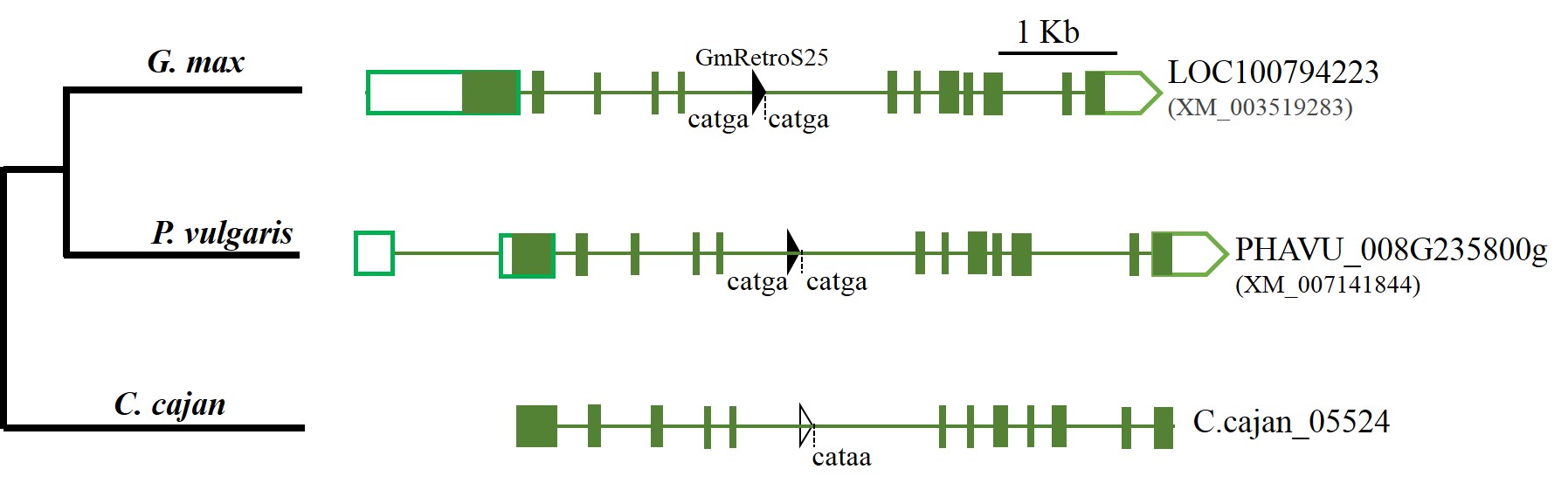
Supplemental Figure S8. A solo-LTR of a TRIM GmaRetroS25 was shared by** **the orthologous genes from** ***G. max*, *P. vulgaris* and *C. cajan*.** Green blocks and lines represent exons and introns, black triangles are complete solo-LTR flaked by 5-bp TSD (catga) and white triangle indicates a truncated solo-LTR. The cDNA sequence for each gene model is shown in ().
